# Acceptor Copolymerized Axially Chiral Conjugated Polymers with TADF Properties for Efficient Circularly Polarized Electroluminescence

**DOI:** 10.1002/advs.202309031

**Published:** 2024-03-29

**Authors:** Wen‐Long Zhao, Ke‐Ke Tan, Wei‐Chen Guo, Chen‐Hao Guo, Meng Li, Chuan‐Feng Chen

**Affiliations:** ^1^ Beijing National Laboratory for Molecular Sciences CAS Key Laboratory of Molecular Recognition and Function Institute of Chemistry Chinese Academy of Sciences Beijing 100190 China; ^2^ School of Chemical Sciences University of Chinese Academy of Sciences Beijing 100049 China; ^3^ College of Chemistry and Chemical Engineering Shanxi University Taiyuan 030006 China

**Keywords:** axial chirality, circularly polarized electroluminescence, conjugated polymers, organic light‐emitting diode, thermally activated delayed fluorescence

## Abstract

Chiral conjugated polymer has promoted the development of the efficient circularly polarized electroluminescence (CPEL) device, nevertheless, it remains a challenge to develop chiral polymers with high electroluminescence performance. Herein, by the acceptor copolymerization of axially chiral biphenyl emitting skeleton and benzophenone, a pair of axially chiral conjugated polymers namely *R*‐**PAC** and *S*‐**PAC** are synthesized. The target polymers exhibit obvious thermally activated delayed fluorescence (TADF) activities with high photoluminescence quantum yields of 81%. Moreover, the chiral polymers display significant circularly polarized luminescence features, with luminescence dissymmetry factor (|*g*_lum_|) of nearly 3 × 10^−3^. By using the chiral polymers as emitters, the corresponding circularly polarized organic light‐emitting diodes (CP‐OLEDs) exhibit efficient CPEL signals with electroluminescence dissymmetry factor |*g*_EL_| of 3.4 × 10^−3^ and high maximum external quantum efficiency (EQE_max_) of 17.8%. Notably, considering both EQE_max_ and |*g*_EL_| comprehensively, the device performance of *R*‐**PAC** and *S*‐**PAC** is the best among all the reported CP‐OLEDs with chiral conjugated polymers as emitters. This work provides a facile approach to constructing chiral conjugated TADF polymers and discloses the potential of axially chiral conjugated luminescent skeletons in architecting high‐performance CP‐OLEDs.

## Graphical abstract

Axially chiral conjugated polymers with thermally activated delayed fluorescence features are synthesized by copolymerizing chiral unit and benzophenone acceptor. By using the obtained polymers as emitters, high‐performance solution‐processed circularly polarized organic light‐emitting diodes are fabricated featuring efficient circularly polarized electroluminescence properties with the maximum external quantum efficiency and dissymmetry factor of up to 17.8% and 3.4 × 10^‐3^, respectively.

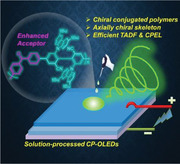

## 1 Introduction

Circularly polarized organic light‐emitting diodes (CP‐OLEDs) have attracted widespread attention owing to the simple device structure and low product cost, as well as their potential application in many fields involving information transmission and high contrast images.^[^ ^1^ ^]^ Thus, various chiral emitting materials have been developed to fabricate highly efficient CP‐OLEDs.^[^ ^2^ ^]^ Wherein, chiral polymers are highly attractive for the superiorities of favorable solubility and morphological stability, as well as molecular diversity, which is conducive to fabricating the CP‐OLEDs feature with flexible structure, large area, and adjustable color.^[^ ^3^ ^]^ Unlike non‐conjugated polymers, chiral conjugated polymers are more promising for obtaining circularly polarized electroluminescence (CPEL) with high efficiency and dissymmetry factor (*g*).^[^ ^4^ ^]^ Since the advent of the first conjugated fluorescent polymer‐based CP‐OLED,^[^ ^5^ ^]^ various chiral conjugated polymers have been designed for fabricating CP‐OLED,^[^ ^6^ ^]^ however, most of them suffered from low device efficiencies. Thus, the development of chiral conjugated polymers with efficient CPEL is an urgent problem to be solved in the next research.

**Figure 1:**
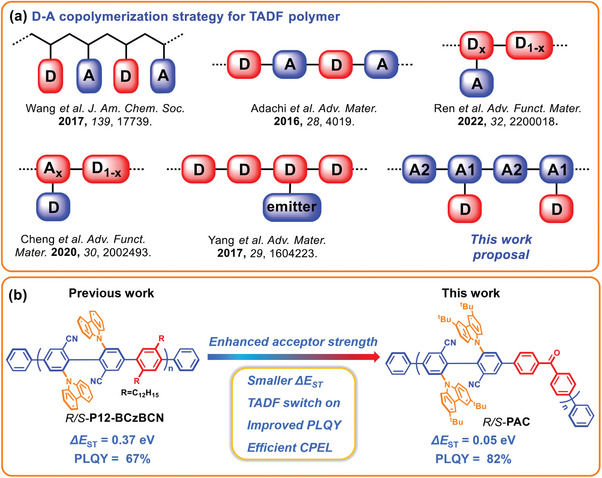
a) D‐A copolymerization strategy for TADF polymer. b) The design of chiral molecular engineering of the axially chiral conjugated polymers for improving their photoelectronic performance.

A variety of strategies have been put forward to pursue all excitons to be effectively used for electroluminescence (EL).^[^ ^7^ ^]^ Among these, thermally activated delayed fluorescence (TADF) materials with a “D‐A” structure are widely considered ideal candidates for the fabrication of efficient OLEDs by utilizing theoretically 100% of both singlet excitons and triplet excitons.^[^ ^8^ ^]^ Currently, conjugated polymers with TADF properties have become a hot spot in OLEDs due to the advantages of molecular diversity and facile realization of highly efficient solution‐processed devices. Although various TADF polymers using D‐A copolymerization strategies have been developed (**Figure** **1**a),^[^ ^9^ ^]^ acceptor copolymerized TADF polymers have not been reported yet. Especially, only a limited number of chiral conjugated polymers with TADF properties have been reported for the enormous challenge of simultaneously achieving effective chirality transfer and efficient luminescence. In 2019, the first chiral TADF polymer was designed by hanging chiral side chains in the polymer main chain,^[^ ^10^ ^]^ and the corresponding device displayed low external quantum efficiency (EQE) of less than 1%, and no CPEL signal was detected. In 2022, our group reported the first case of chiral TADF polymer with CPEL by grafting chiral octa‐hydro‐binaphthol induced acceptors onto the polymer donor main chains,^[^ ^11^ ^]^ and the corresponding CP‐OLEDs exhibited the EQE_max_ of 15.8% and CPEL signals with the |*g*_EL_| values of only 1.6 × 10^−3^. Although chiral conjugated TADF polymers offer the possibility of constructing high‐efficiency CP‐OLEDs, there is still a lot of room for polymers to improve *g*_EL_ values and device efficiencies. The elegant structure design for the chiral conjugated polymer to simultaneously enhance the chiral features and TADF activities is a topic worth further exploration.

To address the above problem, our group previously proposed a method to construct axially chiral TADF molecules with intrinsically chiral emitting skeleton, and the CP‐OLED based on those axially chiral TADF molecules generally displayed high EQE and intense CPEL with large |*g*_EL_| value.^[^ ^12^ ^]^ The dual‐D‐A structure based on biphenyl perfectly integrated chiral core and luminescent groups, allowing chiral features throughout the entire efficiently emissive process.^[^ ^13^ ^]^ More recently, our group reported linear axially chiral conjugated polymers *R/S*‐**P12‐BCzBCN**,^[^ ^14^ ^]^ which were synthesized by copolymerizing axially chiral emitting skeleton with 1,4‐didodecylbenzene units (Figure 1b). The linear polymers exhibited intense circularly polarized luminescence (CPL) features with |*g*_lum_| of 5.2 × 10^−3^ and photoluminescence quantum yield (PLQY) of 66.7%. However, due to the electron delocalization caused by the conjugated elongation of the main chain, the polymers *R/S*‐**P12‐BCzBCN** exhibit a large overlap of frontier molecular orbitals with the energy gap (Δ*E*_ST_) between the lowest singlet excited (S_1_) state and the lowest triplet excite (T_1_) state up to 0.37 eV, thus switching off their TADF characteristics. Recently, reasonably decorating the donors or acceptors around a chiral emitting skeleton has been proven to be an effective design strategy to reduce the Δ*E*_ST_ and improve the quantum yield while maintaining the CPL property.^[^ ^13d,e^ ^]^ Herein, we propose a strategy of acceptor copolymerization to construct axially chiral conjugated polymers *R/S*‐**PAC** with TADF properties (Figure 1b). The stronger electron acceptor benzophenone was introduced to replace the 1,4‐didodecylbenzene as a linker to connect the chiral emitting skeleton unit, and an enhanced acceptor conjugated main chain polymer was obtained. This operation significantly separated the highest occupied molecular orbital (HOMO) and the lowest unoccupied molecular orbital (LUMO), also facilitating the coupling of singlet and triplet states to switch on the TADF activities. As a result, the target chiral conjugated polymers illustrated a tiny Δ*E*_ST_ of 0.05 eV, high PLQYs of 82%, and obvious CPL properties with *g*_lum_ of 3 × 10^−3^. Additionally, the CP‐OLEDs based on *R*‐**PAC** and *S*‐**PAC** exhibited highly efficient CPEL features with |*g*_EL_| value of 3.4 × 10^−3^, as well as EQE_max_ of up to 17.8%. Notably, considering both EQE_max_ and |*g*_EL_| comprehensively, the device performance of *R*‐**PAC** and *S*‐**PAC** is the best among all the reported CP‐OLEDs with chiral conjugated polymers as emitters. Compared with the reported chiral conjugated polymers, *R/S*‐**PAC** exhibited a significant improvement in chiroptical properties and device performance. The result also revealed the copolymerization of skeleton axis chiral luminescent elements and enhanced acceptors was a feasible strategy for constructing efficient chiral conjugated polymers with TADF properties.

## 2 Results and Discussion

### 2.1 Synthesis, Characterization, and Thermal Properties

**Scheme 1:**
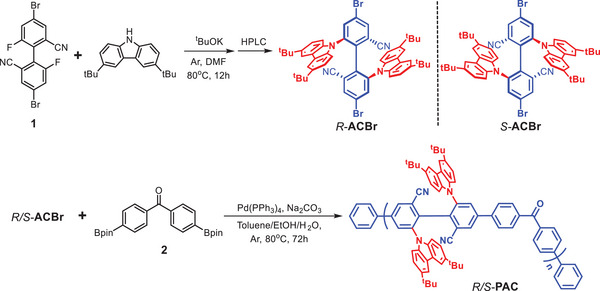
Synthetic routes for axially chiral conjugated polymers *R*‐**PAC** and *S*‐**PAC**.

The synthetic procedures of axially chiral conjugated polymers *R*‐**ACBr** and *S*‐**ACBr** are shown in **Scheme** **1**. Starting from compound **1**, *rac*‐**ACBr** was first prepared through a nucleophilic substitution reaction, followed by a chiral high‐performance liquid chromatography (HPLC) separation to obtain enantiomeric precursor *R*‐**ACBr** and *S*‐**ACBr**. Then, palladium‐catalyzed Suzuki coupling polymerizations were performed between *R*‐**ACBr** or *S*‐**ACBr** with **2** to obtain the target polymers *R*‐**PAC** or *S*‐**PAC**, respectively. The molecular weight and polydispersity index (PDI) measured by gel permeation chromatography (GPC) were 22 kDa and 2.1 for *R*‐**PAC** and 26 kDa and 2.2 for *S*‐**PAC**. The chemical structure of *R*‐**PAC** and *S*‐**PAC** was comprehensively confirmed by ^1^H NMR, and the detailed experimental methods and characterization data are described and listed in the Supporting Information.

To confirm the absolute configuration of enantiomeric monomers, the single crystals of *R*‐**ACBr** and *S*‐**ACBr** which were suitable for X‐ray analysis were obtained by slowly evaporating their dichloromethane/methanol solution. As shown in Figure S1, Supporting Information, the axial chirality of *R*‐**ACBr** and *S*‐**ACBr** was caused by the steric hindrance of the *tert*‐butyl carbazole and cyano units, which prevented the configuration inversion. According to the crystal structure, the biphenyl fragment formed a dihedral angle of nearly 60°, and the twist angles between *tret*‐butyl carbazole and benzonitrile unit were also nearly 60°. The large twist angle between the *tret*‐butyl carbazole donor and biphenyl acceptor was conducive to separating the HOMO and LUMO, thereby reducing Δ*E*_ST_.

Moreover, the configurational stability of *R*‐**ACBr** and *S*‐**ACBr** was also studied. As shown in HPLC analysis (Figures S5 and S6, Supporting Information), both *R*‐**ACBr** and *S*‐**ACBr** displayed no racemization even under the temperature of 373 K for 20 min. The axially chiral monomers with stable configurations are not only beneficial for obtaining good CPL properties but also for constructing chiral conjugated polymers. Furthermore, the target polymers *R*‐**PAC** and *S*‐**PAC** also displayed good thermal stability. According to the thermal analysis results, their decomposition temperature (*T*_d_, at 5% weight loss) was up to 520 °C (see Figures S7 and S8, Supporting Information). The glass transition temperature (*T*_g_) of the polymers was not observed even when the temperature was up to 300 °C (see Figures S9 and S10, Supporting Information), which could be ascribed to their highly rigid and twisted structures. Besides, the introduction of tertiary butyl to chiral polymers can effectively inhibit tight intermolecular π–π stacking, which was conducive to reducing exciton annihilation, as well as improving their solubilities, thus facilitating the preparation of high‐performance solution‐processed CP‐OLEDs.

### 2.2 Theoretical Calculations and Electrochemical Properties

**Figure 2:**
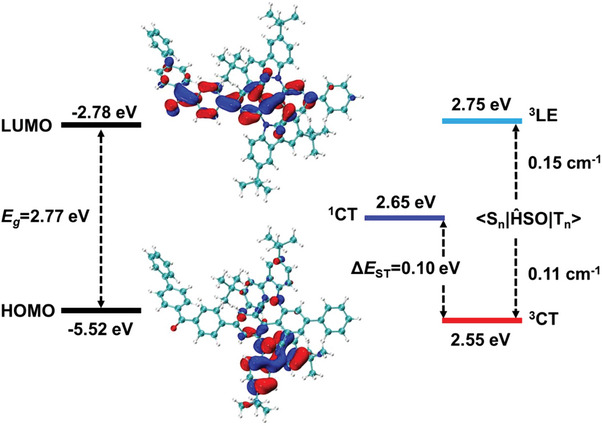
Frontier molecular orbital distribution and energy level of *R*‐**PAC**.

Theoretical calculations were performed to study the geometrical and electronic structures of the target polymers. As shown in **Figure** **2**, the optimized repeating unit structures of the chiral conjugated polymers *R*‐**PAC** were highly twisted, the HOMO was mainly located on the *tert*‐butyl carbazole moieties of *R*‐**PAC**, and the LUMO was predominantly distributed on the acceptor main chain consisting of cyano substituted biphenyls and benzophenone. Compared with *R‐* **P12‐BCzBCN** (Figure S11, Supporting Information), the introduction of benzophenone to *R‐* **PAC** restricted the delocalization degree of the electron cloud, thus causing well‐separated HOMO‐LUMO, as well as a small Δ*E*_ST_ value of 0.1 eV. Moreover, the replacement of the benzophenone linker with the phenyl linker reduced the LUMO energy level of *R‐* **PAC** and formed a small energy gap (*E*_g_) of 2.77 eV. The strong electron‐withdrawing ability of benzophenone red‐shifted the emission spectrum of *R‐* **PAC**.

An in‐depth analysis was conducted on the excited state characteristics of *R‐* **PAC**, and the natural transition orbits (NTOs) were conducted utilizing Multiwfn 3.8.^[^ ^15^ ^]^ The transition characteristics of *S*_1_, *T*_1_, *T*_2_, and *T*_3_ states were investigated, and the small Δ*E*_ST_ (less than 0.11 eV) between *S*_1_ and *T*_n_ was believed to be a potential source of singlet‐triplet coupling.^[^ ^16^ ^]^ As shown in Figure S12, Supporting Information, during the *S*_1_, *T*_1_, and *T*_2_ transition processes, distinctly isolated holes and electrons were respectively located on *tert*‐butyl carbazole donors and main chain acceptors, evidencing the intramolecular charge transfer (ICT) feature of these excited states. Additionally, during the *T*_3_ transition process, both holes and electrons were aggregated on the acceptor fragment composed of biphenyl and benzophenone, which indicated *T*_3_ was a localized excited triplet (^3^LE) state. In general, the reverse intersystem crossing (RISC) process from *T*_1_ to *S*_1_ was a spin‐forbidden transition according to the El‐Sayed rule. Based on the second‐order vibronic coupling mechanism, the ^3^LE state could afford potential supplementary for converting excitons from triplet to singlet state,^[^ ^17^ ^]^ thereby facilitating an efficient RISC process. To verify this inference, the ORCA 5.0.4 package with the B3LYP‐D3(BJ)/6‐31G(d) method was used to calculate the spin‐orbit coupling matrix elements (SOCME) of the different transition channels.^[^ ^18^ ^]^ Finally, the SOCME value of <*S*_1_|ĤSO|*T*_1_> and <*S*_1_|ĤSO|LE_3_> for *S*‐**PAC** was 0.11 and 0.15 cm^−1^, respectively (Figure 2). Thus, assisted by the ^3^LE state with a high value of <*S*_1_|ĤSO|^3^LE>, the chiral conjugated polymer can rapidly convert excitons from triplet to singlet states.

To insight into the electrochemical properties of *R/S*‐**PAC**, the frontier molecular orbital energy levels were studied by cyclic voltammetry. The data are detailed in Figures S13 and S15 and Table S4, Supporting Information. The HOMO energy levels of *R*‐**PAC** and *S*‐**PAC** were calculated to be −5.52 eV according to the oxidation potential curve. The energy gap (*E*_g_) of *R*‐**PAC** and *S*‐**PAC** was estimated to be 2.74 eV according to their UV–vis absorption spectra in toluene (Figures S14 and S16, Supporting Information), Thus, the LUMO energy level of *R*‐**PAC** and *S*‐**PAC** was calculated to be −2.78 eV based on the equation *E*_g_ = *E*_LUMO_ − *E*_HOMO_. By comparing the differences in repetitive units and energy levels between *R‐* **P12‐BCzBCN** and *R*‐**PAC**, it can be roughly deduced that the electrochemical properties of these chiral conjugated polymers can be changed through the replacement of acceptor linkers with different electron‐withdrawing abilities.

### 2.3 Photophysical Properties

**Figure 3:**
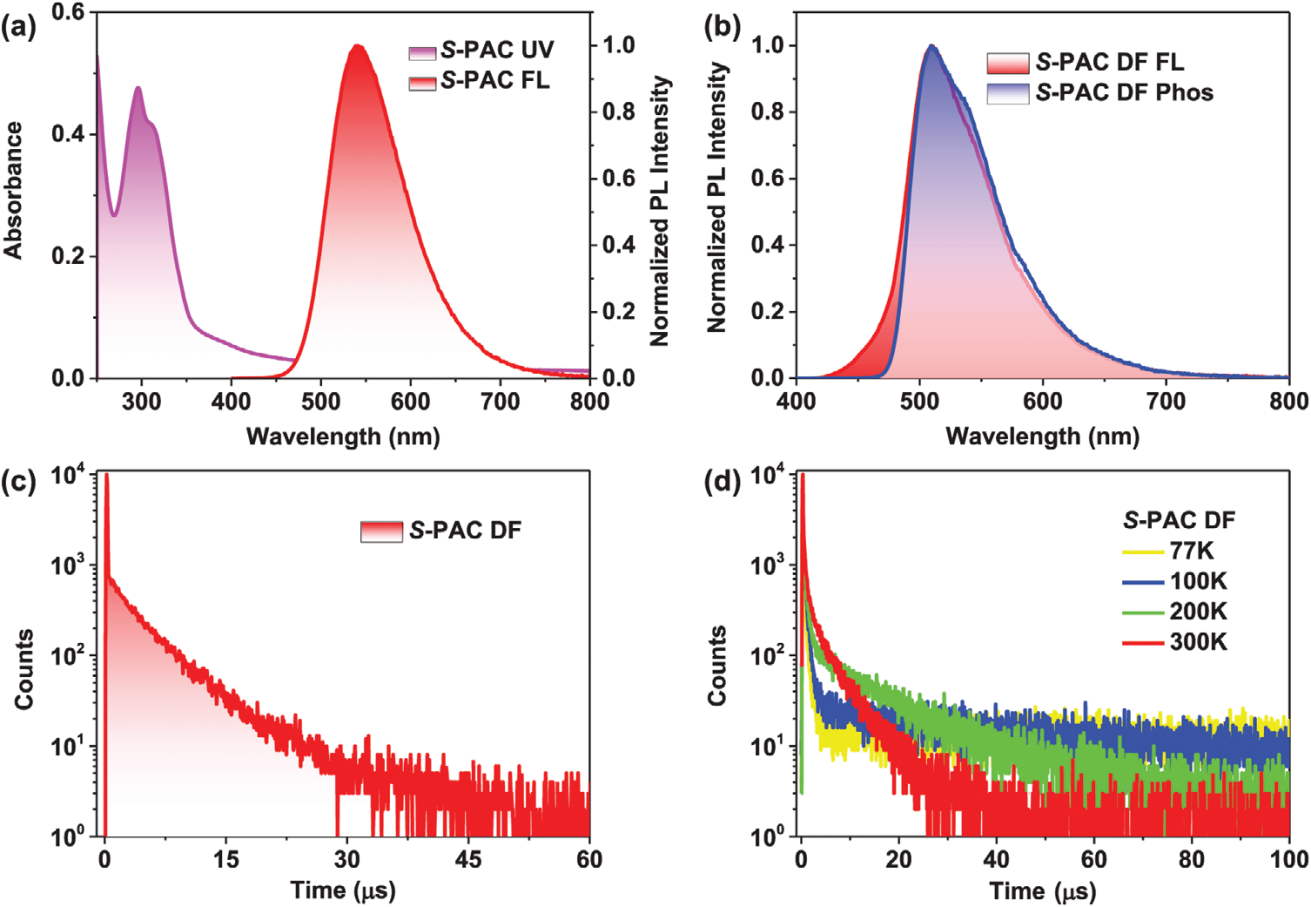
Photophysical properties of *S*‐**PAC**: a) UV–vis absorption and fluorescence spectra at 298 K in neat film. b) Fluorescence and phosphorescence spectra at 77 K in doped film (10 wt% polymer: mCP). c) Transient PL decay curve at 298 K in doped film (10 wt% polymer: mCP). d) Temperature‐dependent transient PL decay curves in doped film (10 wt% polymer: mCP).

The photophysical characteristics of *R*‐**PAC** and *S*‐**PAC** were further studied, the corresponding results of these chiral conjugated polymers were similar, and the relative data of *R*‐**PAC** and *S*‐**PAC** can be found in **Table** **1**. Take *S*‐**PAC** as an example, its UV–vis absorption spectrum in neat film displayed an intense absorption band in short wavelength around 296 nm which could be assigned to the π–π* transition of *S*‐**PAC**, and the comparatively weak and broad absorption ranging from 380 to 420 nm could be attributed to the ICT transition from *tert*‐butyl carbazole to acceptor main chain (**Figure** **3**a). The normalized PL spectrum of *S*‐**PAC** in neat film at 298 K was obtained with an emission band centered at 540 nm. Then, the solvent effect of *S*‐**PAC** in different solvents was also investigated (Figures S18 and S20, Supporting Information). The absorption peaks of *S*‐**PAC** did not occurred obvious movement as the change of solvent environment. The fluorescence spectra of these polymer emitters exhibited apparent bathochromic shift with the increasing solvent polarity, indicative of the presence of the ICT state. The similar photophysical properties of *R*‐**PAC** are shown in Supporting Information. Moreover, the photophysical properties of *S*‐**PAC** in doped films were also tested. 1,3‐di(9H‐carbazol‐9‐yl)benzene (mCP) processes good solubility and high *T*_1_ state level, as well as good film formation properties, which was always selected as a host to fabricate solution‐processed OLED. The doped film of *S*‐**PAC** with a concentration of 10 wt% in mCP illustrated a high PLQY of 81%, which was higher than that of *S‐* **P12‐BCzBCN** (PLQY = 66%). The high PLQY of *S*‐**PAC** provided a strong guarantee for achieving a highly efficient device.

**Table 1:** Summary of photophysical data of *R‐* **PAC** *and S‐* **PAC**.

Compound	*λ*_max_ ^a)^ [nm]	*λ*_max_ ^b)^ [nm]	*Φ*_PL_ ^c)^ [%]	*S*_1_ ^d)^ [eV]	*T*_1_ ^d)^ [eV]	Δ*E*_ST_ ^e)^ [eV]	*τ*_p_ ^f)^ [ns]	*τ*_d_ ^g)^ [µs]	*k*_r_ [10^6^ s^−1^]	*k*_RISC_ [10^5^ s^−1^]
*R*‐**PAC**	541	520	82	2.642	2.594	0.048	41.14	4.68	2.43	2.49
*S*‐**PAC**	542	519	81	2.648	2.594	0.054	40.74	4.65	2.38	2.47

^a)^ Measured in neat films;

^b)^ Doped films (10 wt% polymer: mCP) at 300 K;

^c)^ PLQYs of doped films (10 wt% polymer: mCP);

^d)^ Measured in doped films (10 wt% polymer: mCP) at 77 K;

^e)^ Δ*E*_ST_ = *E*_S1_ − *E*_T1_. Lifetime of;

^f)^ prompt;

^g)^ delayed fluorescence measured in doped films (10 wt% polymer: mCP) at 300 K.

Then, a series of measurements were carried out to study the TADF properties of chiral conjugated polymers, and the detailed data can be found in Table 1, Figure 3, and Supporting Information. Take *S*‐**PAC** as an illustrate, the fluorescence and phosphorescence at 77 K in the doped film were measured (Figure 3b). According to the onset of low‐temperature spectra, the *S*_1_, *T*_1_ energy levels, and Δ*E*_ST_ of *S*‐**PAC** were calculated as 2.64, 2.59, and 0.05 eV, respectively. Moreover, the transient photoluminescence (PL) decay curve of the doped film at 298 K exhibited typically exponential decay behavior (Figure 3c), including a prompt decay component with a lifetime of 41 ns and a delayed decay component with a lifetime of 4.6 µs. The singlet–triplet annihilation (STA) and triplet–triplet annihilation (TTA) processes in the high concentration aggregate state could be effectively inhibited by the shorter delayed lifetime originating from the fast RISC process.^[^ ^19^ ^]^ The TADF properties of *R*‐**PAC** were also studied and are shown in Figures S22 and S23, Supporting Information. The temperature‐dependent transient PL decays of polymer emitters in an mCP host with a doped concentration of 10 wt% were investigated at various temperatures (Figure 3d and Figure S24, Supporting Information). As the temperature increased from 77 to 300 K, the proportion of delay components gradually improved, further demonstrating the TADF characteristics of *R*‐**PAC** and *S*‐**PAC**. Subsequently, the RISC rate constants (*k*_RISC_) of triplet excitons and the radiative transition rate constants (*k*_r_) of singlet excitons were accurately calculated for quantitatively study exciton dynamics in radiative transition processes (Table 1). The *k*_RISC_ values of *R*‐**PAC** and *S*‐**PAC** were nearly 2.5 × 10^5^ s^−1^, and the fast RISC processes played a critical role in highly efficient excited‐state radiative transitions. Additionally, the *k*_r_ was 2.43 × 10^6^ s^−1^ for *R*‐**PAC** and 2.38 × 10^6^ s^−1^ for *S*‐**PAC**, respectively. The high *k*_r_ values of the chiral conjugated polymers were conducive to inhibiting emissive state aggregation and thus preventing exciton quenching.^[^ ^20^ ^]^

### 2.4 Chiroptical Properties

**Figure 4:**
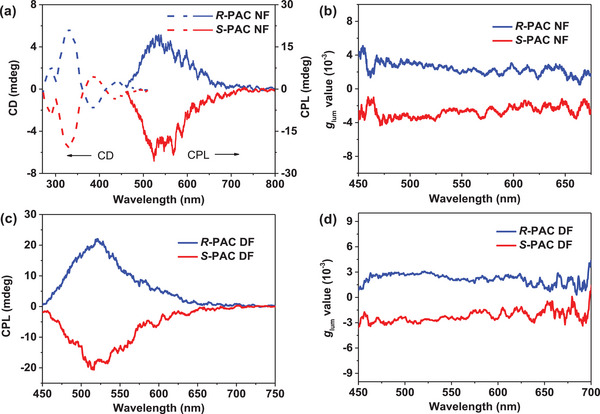
CD and CPL spectra of *R*‐**PAC**/*S*‐**PAC** in a) neat film and c) doped film (10 wt% polymer: mCP); *g*_lum_ versus wavelength curves of *R*‐**PAC**/*S*‐**PAC** in b) neat film and d) doped film.

Circular dichroism (CD) and CPL spectra of *R*‐**PAC** and *S*‐**PAC** were also measured to study their chiroptical properties. As shown in **Figure** **4**a, the two chiral conjugated polymers exhibited almost mirrored CD signals. The intense Cotton effect of *R*‐**PAC** and *S*‐**PAC** in short wavelength regions was attributed to the n–π* and the π–π* electronic transitions of the chiral polymer skeleton, and the relatively weak CD signals ranging from 380 to 440 nm could be assigned to the ICT transition from the *tert*‐butyl carbazole units to the chiral acceptor backbone. Moreover, the CPL spectra of the chiral polymers were then investigated to explore their chiroptical behaviors in excited states. As shown in Figure 4a,b, the chiral conjugated polymers displayed mirror‐imaged CPL signals with *g*_lum_ values of +2.7 × 10^−3^ for *R*‐**PAC** and −2.8 × 10^−3^ for *S*‐**PAC** in neat film, respectively. The CPL signals of *R*/*S*‐**PAC** and *R*/*S*‐**ACBr** in toluene solution were also studied (Figures S25 and S26, Supporting Information), and these chiral compounds exhibited similar CPL intensity and *g*_lum_ values under the same condition. The above result revealed that the strategy of chiral conjugated polymer synthesized by the copolymerization of benzophenone acceptor and chiral unit was beneficial for maintaining the CPL properties of chiral monomers.

In order to study the chiral stability of the chiral conjugated polymers in the device fabricate process, the doped films (10 wt% in mCP) involved in *R*‐**PAC** and *S*‐**PAC** were prepared and then thermal annealed at 60 °C for 20 min. As shown in Figure 4c,d, the CPL spectra of the co‐doped films with *R*‐**PAC** and *S*‐**PAC** were investigated and the corresponding *g*_lum_ values were +2.9 × 10^−3^ and −3.1 × 10^−3^, respectively. The experimental results indicated that the target axially chiral conjugated polymers were ideal candidates for the fabrication of CPEL devices.

### 2.5 Electroluminescence Performances

**Figure 5:**
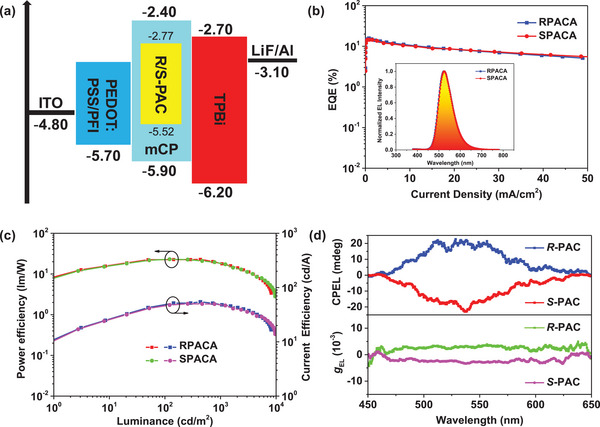
a) Device structures and energy level diagrams of RPACA and SPACA. b) EQE‐current density characteristics of RPACA and SPACA. Inset: EL spectra of devices at 6 V. c) Power efficiency‐luminance‐current efficiency characteristics of RPACA and SPACA. d) The CPEL spectra and *g*_EL_ curves of CP‐OLEDs based on *R*‐**PAC** and *S*‐**PAC**.

Encouraged by their good solubility, high PLQY, and intense CPL, the EL properties of *R*‐**PAC** and *S*‐**PAC** were further investigated. Solution‐processed devices employing the chiral conjugated polymers as emitters were fabricated with the configuration of PEDOT:PSS + PFI (40 nm)/10 wt% polymers: mCP (40 nm)/TPBi (42 nm)/LiF (1 nm)/Al (100 nm). Among them, poly(3,4‐ethylenedioxythiophene):poly‐(styrenesulfonate) (PEDOT:PSS) was mixed with perfluorinated ionomer (PFI) as the hole inject and hole transport layer. 1,3,5‐tris(1‐phenyl‐1H‐benzo[d]imidazol‐2‐yl)benzene (TPBi) was selected as the electron transport layer. The energy level diagrams of these devices and the relational molecular structures are detailed in **Figures** **5**a and S28, Supporting Information. The devices based on *R*‐**PAC** and *S*‐**PAC** were named RPACA and SPACA, respectively. As shown in Figure 5b,c, the device RPACA showed better performances with an EQE_max_ of 15.8%, a maximum current efficiency (CE_max_) of 54.5 cd A^−1^, a power efficiency (PE_max_) of 22.8 lm W^−1^, as well as a *L*_max_ of 8149 cd m^−2^. Moreover, the device SPACA exhibited a CE of 50.3 cd A^−1^, a PE of 22.6 lm W^−1^, an EQE_max_ of 14.5%, and a *L*_max_ of 9661 cd m^−2^, respectively. Both the obtained devices processed a turn‐on voltage of 4.5 V (Figure S27, Supporting Information) and electroluminescence centered at 524 nm. Additionally, the conjugated building blocks enhanced the rigidity of the polymer skeleton and suppressed the energy loss, resulting in a small full width at half maxima of 72 nm. The details of the two devices' performances are summarized in **Table** **2** and Figure S27, Supporting Information.

**Table 2:** The summarized electroluminescence performances of the devices.

Device	*V*_on_ [V]^a)^	*λ*_EL_ [nm]	EQE_max_ [%]^b)^	PE_max_ [lm W^−1^]^c)^	CE_max_ [cd A^−1^]^d)^	*L*_max_ [cd m^−2^]^e)^	FWHM [nm]^f)^	CIE (*x*,*y*)^g)^
RPACA	4.5	524	15.8	22.8	54.5	8149	72	(0.29, 0.61)
SPACA	4.5	524	14.5	22.6	50.3	9661	72	(0.29, 0.61)
RPACB	4	528	17.8	28.3	63	8266	73	(0.31, 0.61)
SPACB	4	528	17.1	31.8	60.6	7966	73	(0.31, 0.61)

^a)^ *V*_on_, the operating voltage at a brightness of 1 cd m^−2^;

^b)^ Maximum external quantum efficiency;

^c)^ Maximum power efficiency;

^d)^ Maximum current efficiency;

^e)^ Maximum luminance;

^f)^ Full width at half maxima;

^g)^ Commission Internationale de I'Eclairage 1931 coordinates.

In order to further improve the EL performance of the chiral conjugated polymer, 4,4′‐di(9H‐carbazol‐9‐yl)‐1,1′‐biphenyl (CBP) was selected as host material to fabricate devices with the following architecture (devices RPACB and SPACB): PEDOT:PSS + PFI (40 nm)/10 wt% emitters: CBP (40 nm)/TPBi (42 nm)/LiF (1 nm)/Al (100 nm). The optimized device structure and the energy levels can be found in Figure S28, Supporting Information. The device's performance is detailed in Table 2. Both the doped devices processed a low turn‐on voltage of 4 V (Figure S31, Supporting Information), which was lower than that of mCP‐based ones. Moreover, the device RPACB exhibited a high *L*_max_ of 8266 cd m^−2^, and EQE_max_, PE_max_, and CE_max_ values were 17.8%, 63 lm W^−1^, and 28.3 cd A^−1^ (Figures S29 and S32, Supporting Information), respectively. As far as we know, the EQE_max_ of 17.8% is the highest value among all reported chiral conjugated polymers (Table S5, Supporting Information).

Subsequently, the CPEL activities of *R*‐**PAC** and *S*‐**PAC** were further evaluated. As depicted in Figure 5d, nearly mirrored CPEL spectra accompanied with opposing *g*_EL_ signals were detected from the chiral conjugated polymer‐based CP‐OLEDs. The *g*_EL_ values were +3.2 × 10^−3^ for *R*‐**PAC** and −3.4 × 10^−3^ for *S*‐**PAC**, respectively. Based on the above experimental results, it can be concluded that the axially chiral repeating unit played a key role in the processes of CPL and CPEL. Additionally, the copolymerization of the chiral conjugated emitting skeleton and conjugated acceptor not only enhanced the EL behavior but also achieved efficient CPEL. Besides, the satisfactory results also revealed the broad prospects of the axially chiral biphenyl emitter in the construction of highly efficient chiral conjugated polymer through the strategy of acceptor copolymerization.

## 3 Conclusion

In summary, we propose a strategy of acceptor copolymerization to construct axially chiral conjugated polymers with TADF properties. The target chiral polymers *R*‐**PAC** and *S*‐**PAC** were synthesized by copolymerizing benzophenone with axially chiral biphenyl. The polymers possessed chiral conjugated main chains and obviously separated HOMO and LUMO, leading to obvious TADF features with small Δ*E*_ST_ of 0.05 eV and high PLQYs of 81%. Moreover, the intense chiroptical activities were detected by CD and CPL spectra with the |*g*_lum_| values of nearly 3 × 10^−3^ in film states. Besides, the chiral conjugated polymers not only achieved a remarkable EQE_max_ of 17.8% but also displayed intense CPEL signals with |*g*_EL_| of 3.4 × 10^−3^. It is worth pointing out that considering both EQE_max_ and |*g*_EL_| comprehensively, the device performance of *R*‐**PAC** and *S*‐**PAC** is the best among all the reported CP‐OLEDs with chiral conjugated polymers as emitters. The excellent performance of *R*‐**PAC** and *S*‐**PAC** evidenced that the copolymerization of the chiral conjugated emitting skeleton and conjugated acceptor is an effective method for constructing high‐performance chiral conjugated TADF polymer, also providing a new viewpoint for the achievement of highly efficient CPEL based on chiral polymers.

## Conflict of Interest

The authors declare no conflict of interest.

## Supporting information

Supporting Information

Supporting Information

## Data Availability

The data that support the findings of this study are available in the supplementary material of this article.

## References

[1] a) R. Farshchi , M. Ramsteiner , J. Herfort , A. Tahraoui , H. T. Grahn , Appl. Phys. Lett. 2011, 98, 162508;

[2] a) L. Xu , H. Liu , X. Peng , P. Shen , B. Z. Tang , Z. Zhao , Angew. Chem., Int. Ed. 2023, 62, e202300492; 10.1002/anie.202300492 36825493

[3] a) Y.‐F. Wang , M. Li , J.‐M. Teng , H.‐Y. Zhou , W.‐L. Zhao , C.‐F. Chen , Angew. Chem., Int. Ed. 2021, 60, 23619; 10.1002/anie.202110794 34490710

[4] a) Y. Geng , A. Trajkovska , S. W. Culligan , J. J. Ou , H. M. P. Chen , D. Katsis , S. H. Chen , J. Am. Chem. Soc. 2003, 125, 14032; 14611239 10.1021/ja037733e

[5] E. Peeters , M. P. T. Christiaans , R. A. J. Janssen , H. F. M. Schoo , H. P. J. M. Dekkers , E. W. Meijer , J. Am. Chem. Soc. 1997, 119, 9909.

[6] D.‐W. Zhang , M. Li , C.‐F. Chen , Chem. Soc. Rev. 2020, 49, 1331. 31999286 10.1039/c9cs00680j

[7] a) S. Y. Byeon , D. R. Lee , K. S. Yook , J. Y. Lee , Adv. Mater. 2019, 31, 1803714; 10.1002/adma.201803714 30761642

[8] a) Z. Yang , Z. Mao , Z. Xie , Y. Zhang , S. Liu , J. Zhao , J. Xu , Z. Chi , M. P. Aldred , Chem. Soc. Rev. 2017, 46, 915; 28117864 10.1039/c6cs00368k

[9] a) S. Y. Lee , T. Yasuda , H. Komiyama , J. Lee , C. Adachi , Adv. Mater. 2016, 28, 4019; 27001891 10.1002/adma.201505026

[10] Y. Hu , F. Song , Z. Xu , Y. Tu , H. Zhang , Q. Cheng , J. W. Y. Lam , D. Ma , B. Z. Tang , ACS Appl. Polym. Mater. 2019, 1, 221.

[11] J.‐M. Teng , D.‐W. Zhang , Y.‐F. Wang , C.‐F. Chen , ACS Appl. Mater. Interfaces 2022, 14, 1578. 34962755 10.1021/acsami.1c20244

[12] M. Li , Y.‐F. Wang , D. Zhang , L. Duan , C.‐F. Chen , Angew. Chem., Int. Ed. 2020, 59, 3500. 10.1002/anie.201914249 31872521

[13] a) Y.‐F. Wang , M. Li , W.‐L. Zhao , Y.‐F. Shen , H.‐Y. Lu , C.‐F. Chen , Chem. Commun. 2020, 56, 9380; 10.1039/d0cc03822a 32672776

[14] D.‐W. Zhang , M. Li , C.‐F. Chen , Angew. Chem., Int. Ed. 2022, 61, e202213130. 10.1002/anie.202213130 36175371

[15] a) R. L. Martin , J. Chem. Phys. 2003, 118, 4775;

[16] Y. Wang , J. Yang , Y. Gong , M. Fang , Z. Li , B. Z. Tang , SmartMat 2020, 1, e1006.

[17] a) M. K. Etherington , J. Gibson , H. F. Higginbotham , T. J. Penfold , A. P. Monkman , Nat. Commun. 2016, 7, 13680; 27901046 10.1038/ncomms13680 PMC5141373

[18] a) K. R. Naveen , H. Lee , L. H. Seung , Y. H. Jung , C. P. Keshavananda Prabhu , S. Muruganantham , J. H. Kwon , Chem. Eng. J. 2023, 451, 138498;

[19] T. Hosokai , H. Matsuzaki , H. Nakanotani , K. Tokumaru , T. Tsutsui , A. Furube , K. Nasu , H. Nomura , M. Yahiro , C. Adachi , Sci. Adv. 2017, 3, e1603282. 28508081 10.1126/sciadv.1603282 PMC5425233

[20] X. Tong , Z. Zhao , L. Hua , Y. Zhang , B. Xu , Y. Liu , S. Yan , Z. Ren , Adv. Funct. Mater. 2023, 33, 2305324.

