# Supplementary material for: Acceptor Copolymerized Axially Chiral Conjugated Polymers with TADF Properties for Efficient Circularly Polarized Electroluminescence
Source: Adv Sci (Weinh). 2024 Mar 29;11(23):2309031. doi: 10.1002/advs.202309031 (PMC11186117; doi:10.1002/advs.202309031)
Supplement: Supplementary file 1 — Supporting Information [file ADVS-11-2309031-s001.pdf]

ADVANCED SCIENCE
Open Access

Supporting Information

for *Adv. Sci.*, DOI 10.1002/advs.202309031

Acceptor Copolymerized Axially Chiral Conjugated Polymers with TADF Properties for Efficient Circularly Polarized Electroluminescence

Wen-Long Zhao, Ke-Ke Tan, Wei-Chen Guo, Chen-Hao Guo, Meng Li and Chuan-Feng Chen**

Supporting Information

Acceptor Copolymerized Axially Chiral Conjugated Polymers with TADF Properties for Efficient Circularly Polarized Electroluminescence

Wen-Long Zhao, Ke-Ke Tan, Wei-Chen Guo, Chen-Hao Guo, Meng Li and Chuan-Feng Chen**

Contents

1. **General Information--S2**
2. **Experimental Section---S3**
3. **X-Ray Crystal Structure Analysis--S5**
4. **Chiral HPLC Data---S6**
5. **Thermal Properties---S9**
6. **Theoretical Calculations and Electrochemical Properties----------S11**
7. **Photophysical Properties---S14**
8. **Device Fabrication and Characterization--------------------------------S19**
9. **Copies of ^{1}H NMR and ^{13}C NMR Spectra of New Compounds-----S23**
10. **References---S27**

1. General Information

All reagents were purchased from commercial providers without further purification. ^{1}H NMR and ^{13}C NMR spectra were recorded on AVIII 500 MHz NMR spectrometers in $CDCl_3$ solutions. High-resolution mass spectra were measured on a Thermo Fisher® Exactive high resolution LC-MS spectrometer. HPLC analysis were performed on Agilent 1260 Infinity. Analytical injections were performed on chiral stationary phase using the column (Chiralpak® ID, 4.6 mm × 150 mm) and the mobile phase of methanol. Single crystal data was obtained on a Bruker Smart APEXII CCD diffractometer using graphite monochromated Cu Kα radiation. The average weights of the polymers were measured by gel permeation chromatography (GPC) on Waters 410 using polystyrene as standard and THF eluent. The thermogravimetric analysis (TGA) and differential scanning calorimetric (DSC) measurement were respectively performed on Q600 thermogravimeter and Q2000 simultaneous thermal analyzer at a heating rate of 10 °C min^{-1} in nitrogen. Cyclic voltammetry was performed using a CHI600A analyzer with a scan rate of 100 mV/s at room temperature. A conventional three electrode cell was used as electrolytic cell with a glassy carbon working electrode, Pt wire as the counter electrode, and an Ag/Ag^+ (0.01 M $AgNO_3$) as the reference electrode. The oxidation potential was measured in dichloromethane with 0.1 M of tetra-*n*-butylammonium hexafluorophosphate (n-Bu_4NPF_6) as a supporting electrolyte. Ferrocene used as internal standard for calibrating the reference electrode.

The repetitive unit structure of the chiral polymer was optimized with dispersion corrected density functional theory (DFT-D3) at the B3LYP-D3(BJ)/6-31G(d) level using Gaussian 09 program.[S1] The energy levels of excited states were calculated at the B3LYP-D3(BJ)/6-31G(d) level with the time-dependent density functional theory (TD-DFT) method. The natural transition orbits (NTOs) were calculated through TD-DFT method and the Multiwfn 3.8 program was used to analyze the characteristics of excited states. The spin-orbit coupling (SOC) matrix elements were calculated using the spin-orbit mean-field (SOME) methods based on the excited state wave functions obtained from TD-DFT calculations. All these calculations were performed utilizing ORCA 5.0.4 program. The visualization of the frontier molecular orbitals was rendered using Visual Molecular Dynamic program (VMD).[S2]

UV-Vis spectra were recorded on PerkinElmer® UV/Vis/NIR spectrometer (Lambda 950). The photoluminescence spectra and transient PL decay characteristics were measured on an Edinburgh Instruments FLS 1000 spectrometer. The absolute photoluminescence quantum yield (PLQY) was measured on the FLS 1000 spectrometer using an integrating sphere at an

excitation wavelength of 340 nm. The circular dichroism (CD) spectra were recorded on a JASCO J810 spectropolarimeter. The circularly polarized photoluminescence (CPL) and circularly polarized electroluminescence (CPEL) measurements were performed utilizing a commercialized instrument JASCO CPL-300 spectrophotometer at room temperature.

2. Experimental Section

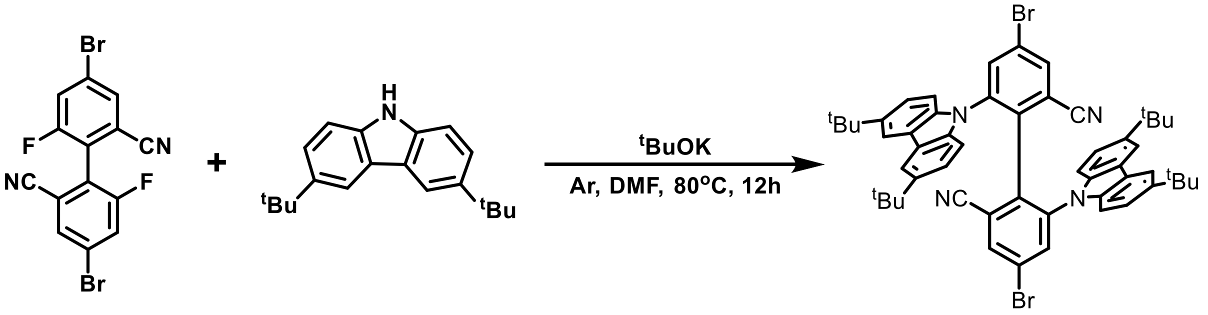

1 *rac*-**ACBr**

Synthesis of ***(6r,6's)-4,4'-dibromo-6,6'-bis(3,6-di-tert-butyl-9H-carbazol-9-yl)-[1,1'-biphenyl]-2,2'-dicarbonitrile*** **(*rac*-ACBr)**: Under argon atmosphere, 3,6-di-tert-butyl-9H-carbazole (3.4 g, 12 mmol) in anhydrous DMF (35 mL) was added dropwise into an anhydrous DMF (20 mL) solution containing ^{t}BuOK (1.5 g, 13 mmol) within 15 min and then stirred for 1 h at room temperature. Subsequently, compound **1** (2.0 g, 5 mmol) was dissolved in the anhydrous DMF (10 mL) and added dropwise into above system within 15 min, then the final mixture was continue stirred at 80 °C for 12 h. After that, the mixture was poured into water (200 mL), and extracted with ethyl acetate (3×50 mL). The combined organic layer was washed with saturated brine (3×50 mL), dried over anhydrous $MgSO_4$, and then concentrated under reduced pressure. The residue was purified by column chromatography with petroleum ether/dichloromethane (1:1, v/v) as eluent to give *rac*-**ACBr** (3.6 g, yield: 78 %) as white solid. ^{1}H NMR (500 MHz, $CDCl_3$): δ 8.08 (s, 2H), 7.87 (s, 2H), 7.63 (s, 2H), 7.38 (s, 2H), 7.31 (d, J = 8.6 Hz, 2H), 6.89 (d, J = 8.6 Hz, 2H), 6.63 (d, J = 8.6 Hz, 2H), 5.54 (d, J = 8.6 Hz, 2H), 1.43 (s, 18H), 1.32 (s, 18H); ^{13}C NMR (126 MHz, $CDCl_3$): δ 144.1, 143.6, 141.5, 140.3, 139.4, 137.4, 135.6, 134.9, 124.8, 124.7, 124.4, 123.6, 123.2, 118.4, 116.8, 116.7, 115.4, 109.8, 108.9, 34.7, 34.5, 31.9. HR-MS (APCI): *m/z* calcd. For $C_{54}H_{52}N_2Br_4$ $[M+H]^+$ 917.2650, found 917.2614.

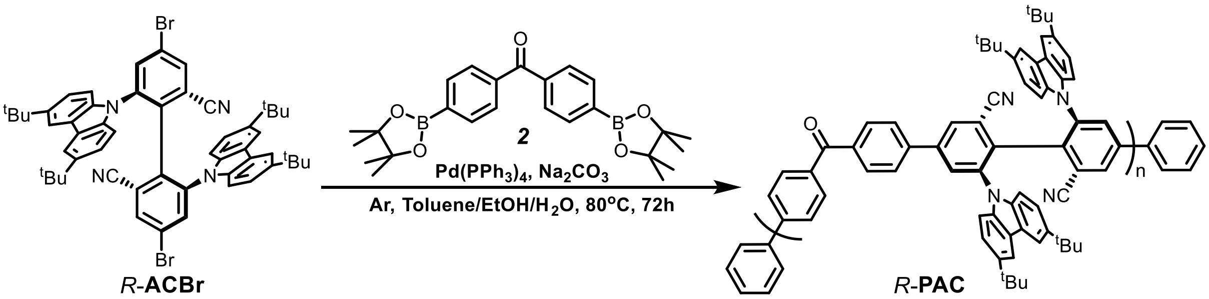

R-**ACBr** *R*-**PAC**

Synthesis of *R*-**PAC**: A mixture of *R*-**ACBr** (165 mg, 0.18 mmol), compound **2** (78 mg, 0.18 mmol), $Pd(PPh_3)_4$ (21 mg, 0.018 mmol), and Na_2CO_3 (0.19 g, 1.8 mmol) was added to a mixture solvent of 4 mL toluene, 2 mL ethanol and 1 mL water under argon atmosphere. The resulting mixture was stirred at 80 °C for 72 h. Then, benzene boronic acid (24 mg, 0.20 mmol) in toluene (0.5 mL) was added into the reaction system, and the mixture was subsequently stirred for another 12 h at 80 °C. Then, bromobenzene (31 mg, 0.20 mmol) was added into the reaction system, and the mixture was continue stirred for 12 h at 80 °C. Then, sodium diethyldithiocarbamate trihydrate (8 mg, 0.036 mmol in 1 mL H_2O) was added and stirred at 80°C for 12 h. After cooling the reaction system to room temperature, the mixture was poured into water and the organic phase was extracted with dichloromethane. The organic layer was filtered through a short kieselguhr, and then concentrated under reduced pressure. Then the crude product was concentrated in 2 ml of dichloromethane and precipitated in methanol (300 mL). After filtering out the sediment, further purification could be performed by Soxlet extraction with acetone at 85 °C for 24 h and dried via vacuum to afford the final product *R*-**PAC** as a green solid (115.8 mg, 68%). ^1H NMR (500 MHz, $CDCl_3$): δ 8.18 (s, 2H), 7.78–7.68 (m, 6H), 7.55–7.44 (m, 8H), 7.22 (s, 4H), 6.92 (d, J = 8.4 Hz, 2H), 6.57 (d, J = 7.9 Hz, 2H), 5.52 (d, J = 8.5 Hz, 2H), 1.32 (s, 18H), 1.25 (s, 18H). GPC (THF, polystyrene standard) analysis showed a M_w = 21961 and PDI = 2.1.

The synthesis procedure of *S*-**PAC** was similar to that of the *R*-**PAC**. The final product of *S*-**PAC** was a green solid (101.6 mg, 60%). ^1H NMR (500 MHz, $CDCl_3$): δ 8.18 (s, 2H), 7.79–7.68 (m, 6H), 7.55–7.44 (m, 8H), 7.29–7.14 (m, 4H), 6.92 (d, J = 8.5 Hz, 2H), 6.57 (d, J = 8.2 Hz, 2H), 5.52 (d, J = 8.5 Hz, 2H), 1.32 (s, 18H), 1.25 (s, 18H). GPC (THF, polystyrene standard) analysis showed a M_w = 25867 and PDI = 2.2.

3. X-Ray Crystal Structure Analysis

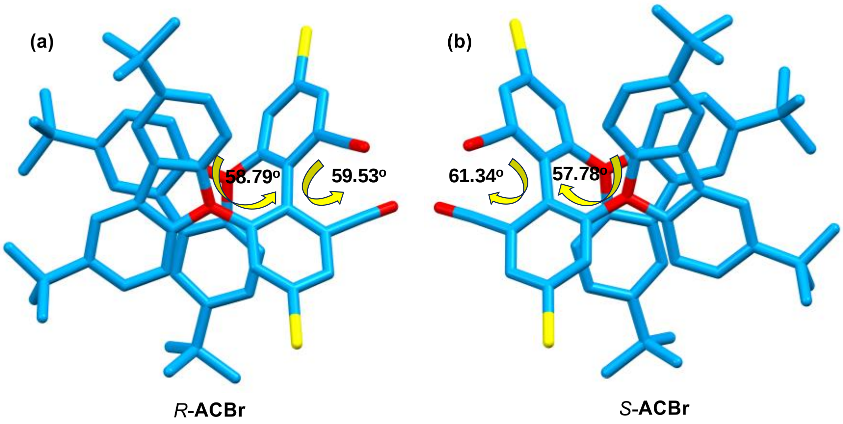

Figure S1. Crystal structure of a) *R*-**ACBr** and b) *S*-**ACBr**.

Table S1. Crystal data and structure refinement for *R*-**ACBr**.

CCDC	2264881
Empirical formula	$C_{54}H_{52}Br_2N_4$
Formula weight	916.81
Temperature/K	169.99(10)
Crystal system	monoclinic
Space group	$P2_1$
a/Å	12.50070(10)
b/Å	15.59217(11)
c/Å	12.89385(10)
α /°	90
β /°	95.4592(7)
γ /°	90
Volume/Å3	2501.78(3)
Z	2
ρ calcg/cm3	1.217
μ /mm−1	2.333
F(000)	948.0
Crystal size/mm3	0.06 × 0.04 × 0.04
Radiation	CuKα (λ = 1.54184)
2Θ range for data collection/°	6.886 to 151.746
Index ranges	-15 ≤ h ≤ 15, -18 ≤ k ≤ 19, -14 ≤ l ≤ 16
Reflections collected	46704
Independent reflections	9951 [Rint = 0.0441, Rsigma = 0.0287]
Data/restraints/parameters	9951/34/593
Goodness-of-fit on F2	1.041
Final R indexes [I>=2σ (I)]	R_1 = 0.0603, wR_2 = 0.1812
Final R indexes [all data]	R_1 = 0.0615, wR_2 = 0.1829
Largest diff. peak/hole / e Å-3	2.53/-1.13

Table S2. Crystal data and structure refinement for *S*-**ACBr.**

CCDC	2264900
Empirical formula	$C_{54}H_{52}Br_2N_4$
Formula weight	916.81
Temperature/K	169.99(10)
Crystal system	monoclinic
Space group	$P2_1$
a/Å	12.47560(10)
b/Å	15.6189(2)
c/Å	12.9645(2)
α /°	90
β /°	95.1020(10)
γ /°	90
Volume/Å3	2516.19(5)
Z	2
ρ calcg/cm3	1.210
μ /mm−1	2.319
F(000)	948.0
Crystal size/mm3	0.06 × 0.04 × 0.04
Radiation	CuKα (λ = 1.54184)
2Θ range for data collection/°	6.846 to 151.038
Index ranges	-15 ≤ h ≤ 14, -19 ≤ k ≤ 19, -16 ≤ l ≤ 16
Reflections collected	36187
Independent reflections	10102 [Rint = 0.0462, Rsigma = 0.0290]
Data/restraints/parameters	10102/1/554
Goodness-of-fit on F2	1.549
Final R indexes [I>=2σ (I)]	R_1 = 0.1002, wR_2 = 0.3069
Final R indexes [all data]	R_1 = 0.1009, wR_2 = 0.3094
Largest diff. peak/hole / e Å-3	5.25/-0.59

4. Chiral HPLC Data

Chiral HPLC has been used in optical resolution of *rac*-**ACBr** to obtain the axial enantiomers *S*-**ACBr** and *R*-**ACBr**, and the HPLC resolution conditions are shown as below: Column: Chiralpak® IC, 2.5cm I.D. × 25 cm L; Mobile phase: *n*-Hexane/DCM=60/40（v/v); Flow rate: 40mL/min; Wavelength: UV 254nm.

HPLC Analysis Conditions:

Column: Chiralpak® ID, 4.6 mm × 150 mm

Mobile phase: MeOH = 100 %

Flow rate: 0.7 mL/min

Abs. detector: 254 nm

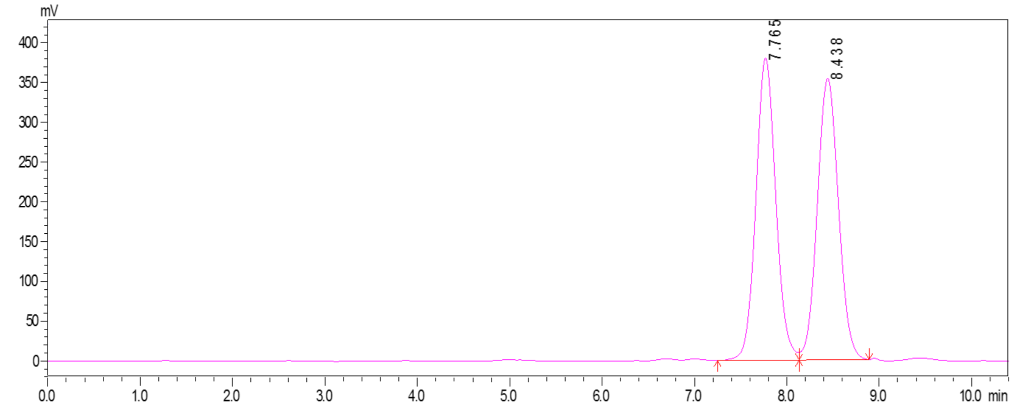

Figure S2. HPLC profile of *rac*-**ACBr**.

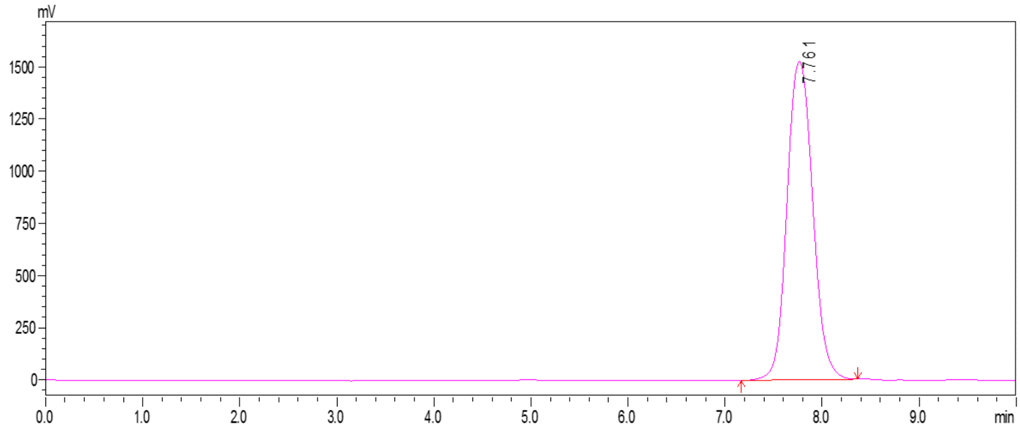

Figure S3. HPLC profile of *R*-**ACBr**.

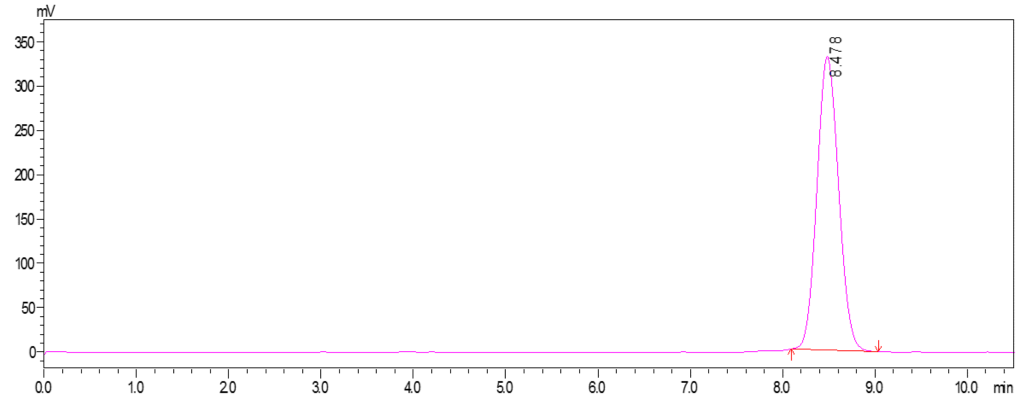

Figure S4. HPLC profile of *S*-**ACBr**.

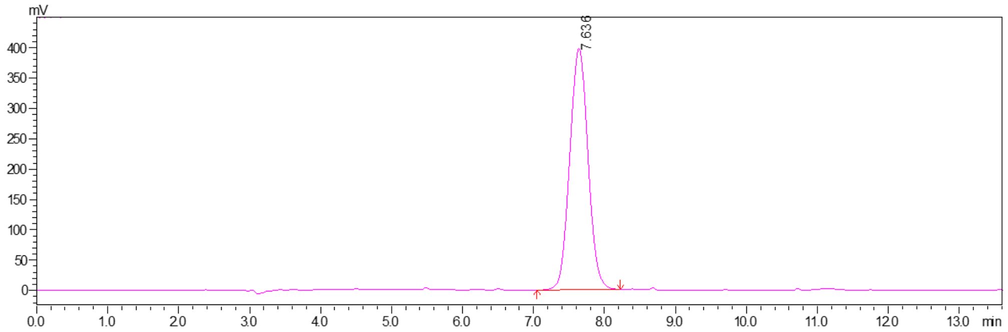

Figure S5. HPLC profile of *R*-**ACBr** after heating to 100ºC.

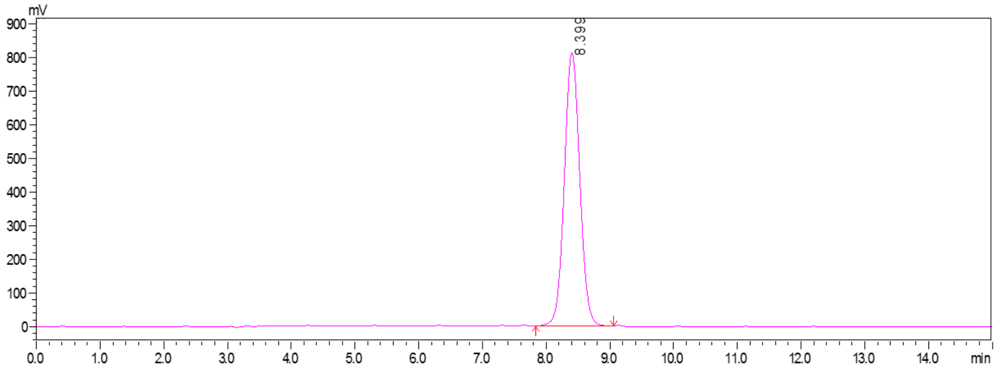

Figure S6. HPLC profile of *S*-**ACBr** after heating to 100ºC.

Table S3. The summary of HPLC profiles of *rac*-**ACBr** and *R/S*-**ACBr**.

Compound	RetTime (min)	Height (mAU)	Area (mAU·s)	Area (%)	ee Value
rac-**ACBr**	7.765	38.0188	571.3393	50.4265	-
	8.438	35.0143	561.6747	49.5735	-
R-**ACBr**	7.761	15.2581	281.04028	100	> 99%
	-	-	-	-	
S-**ACBr**	8.478	33.0990	540.4190	100	> 99%
	-	-	-	-	

5. Thermal Properties

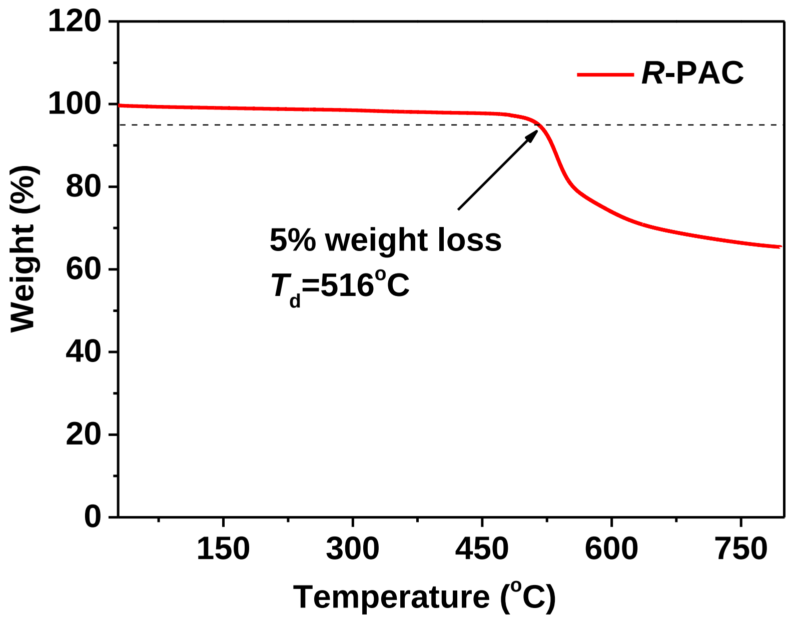

Figure S7. TGA thermogram of *R*-**PAC** recorded under nitrogen at a heating rate of 10 °C/min.

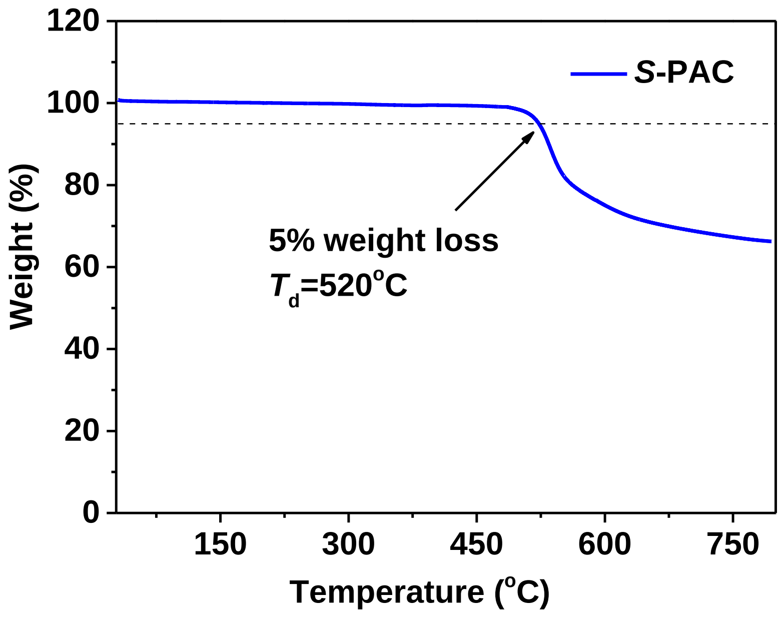

Figure S8. TGA thermogram of *S*-**PAC** recorded under nitrogen at a heating rate of 10 °C/min.

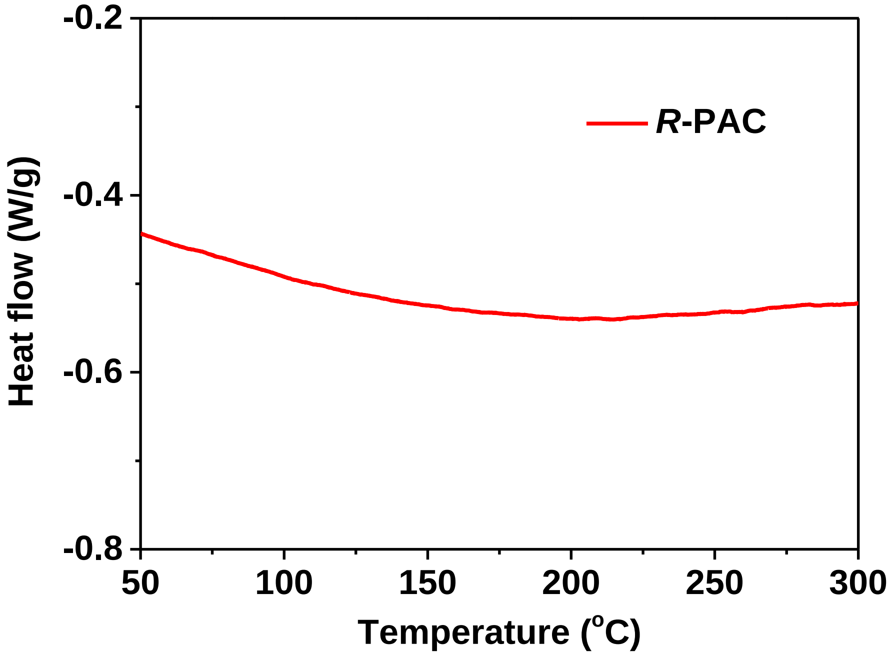

Figure S9. DSC curve of *R*-**PAC** recorded under nitrogen at a heating rate of 10 °C/min.

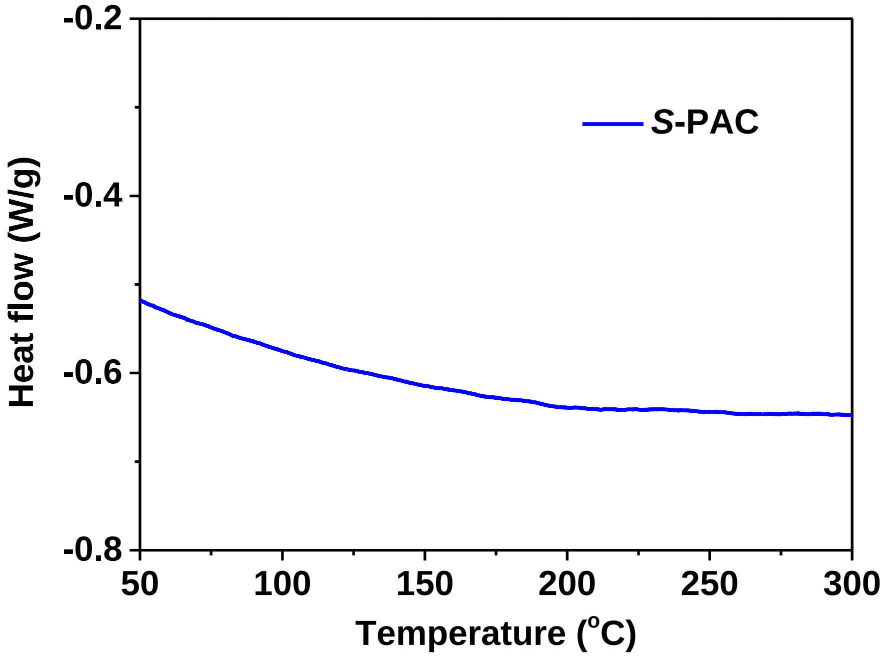

Figure S10. DSC curve of *S*-**PAC** recorded under nitrogen at a heating rate of 10 °C/min.

6. Theoretical Calculations and Electrochemical Properties

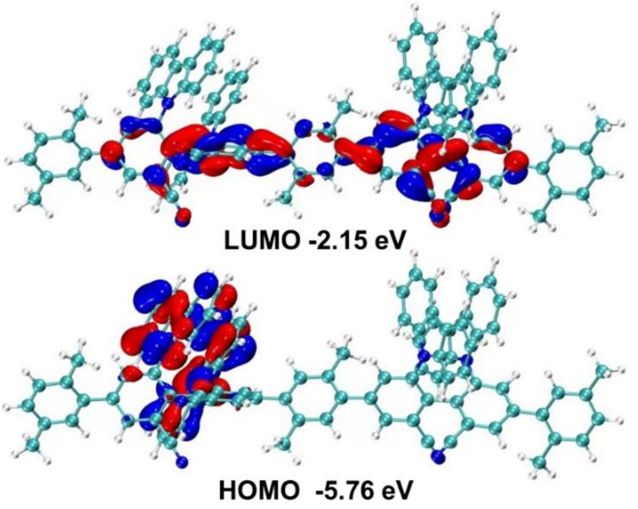

Figure S11. Frontier molecular orbital distribution and energy level of *R*-**P12-BCzBCN**.

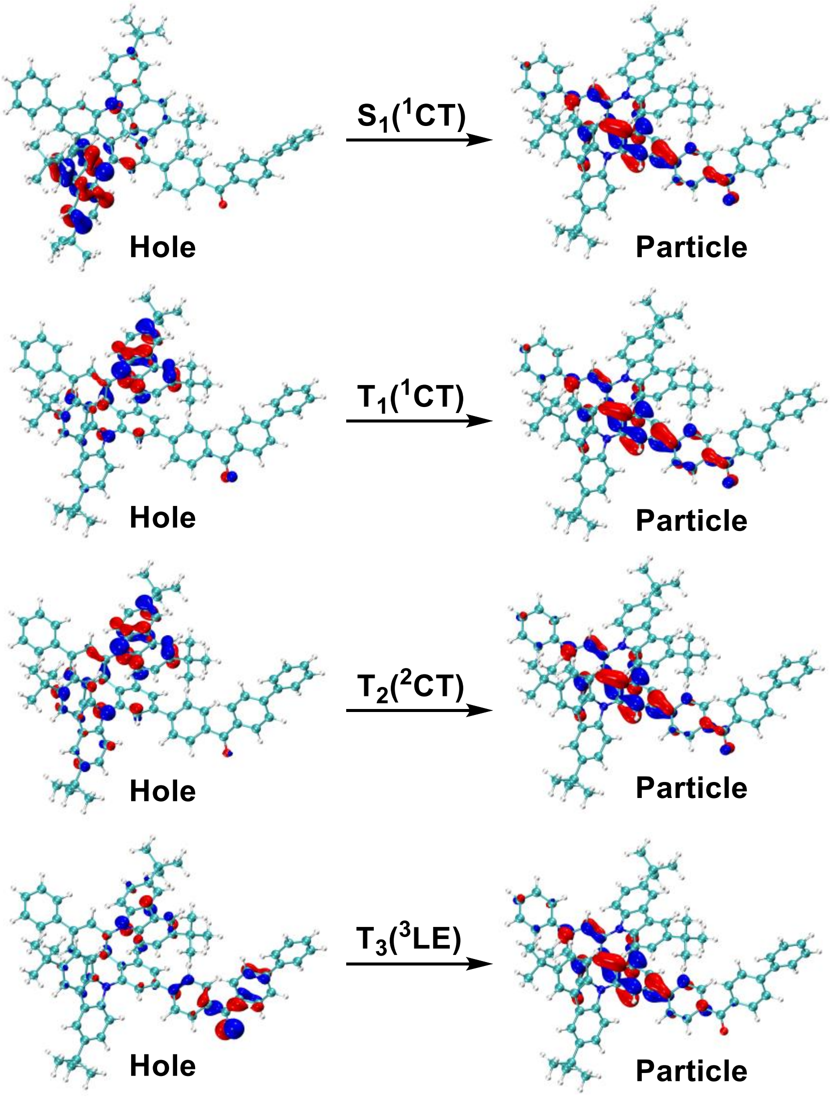

Figure S12. Natural transition orbitals of S_1 and T_n (n = 1, 2) excited states of *S*-**PAC**.

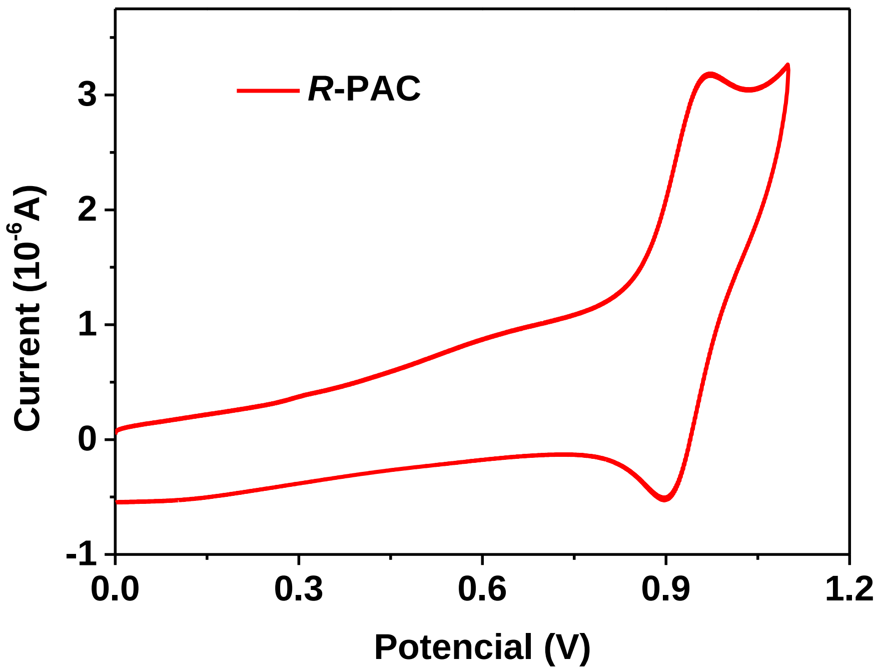

Figure S13. Cyclic voltammograms of *R*-**PAC** measured in dichloromethane.

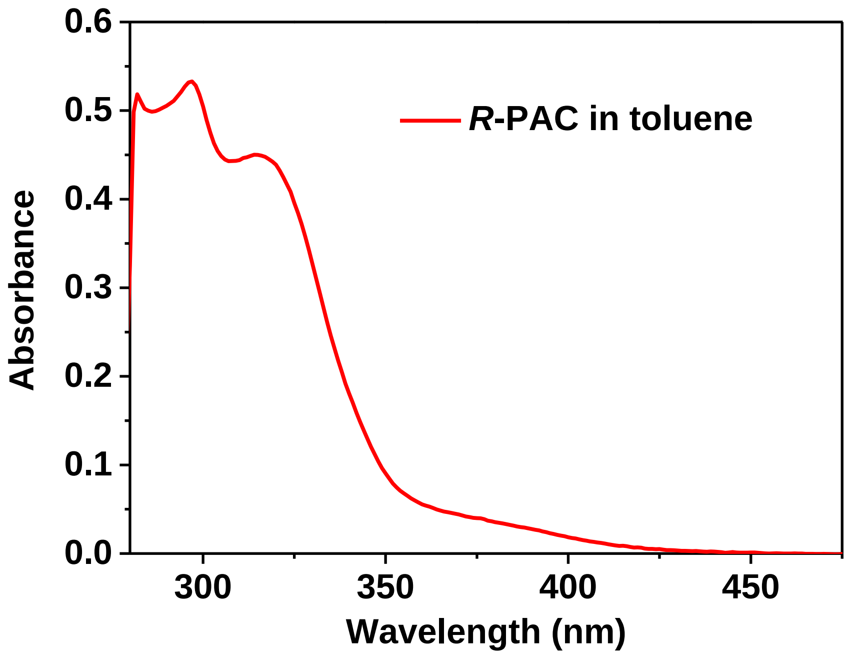

Figure S14. UV-Vis absorption of *R*-**PAC** spectra in toluene (c =1 × 10^{-5} M).

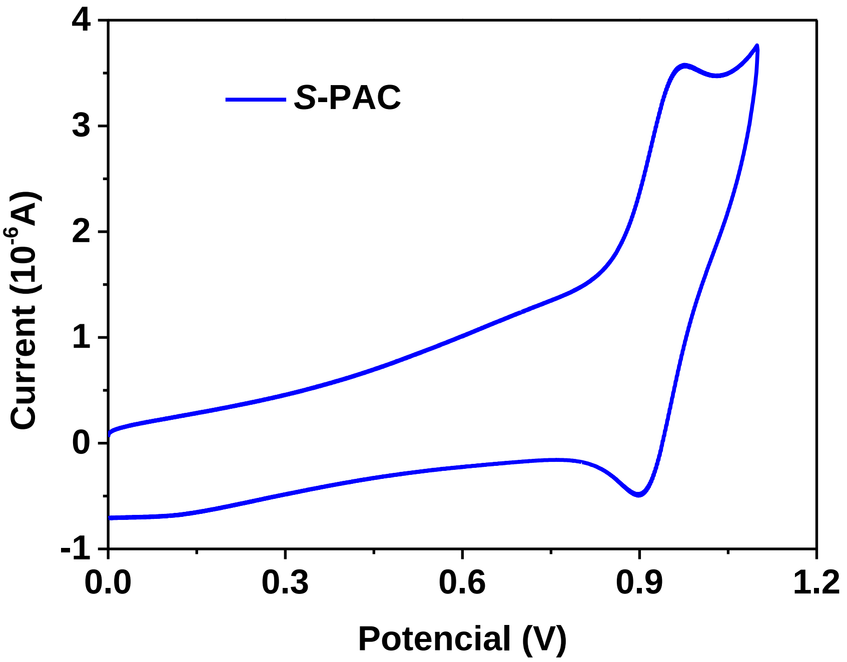

Figure S15. Cyclic voltammograms of *S*-**PAC** measured in dichloromethane.

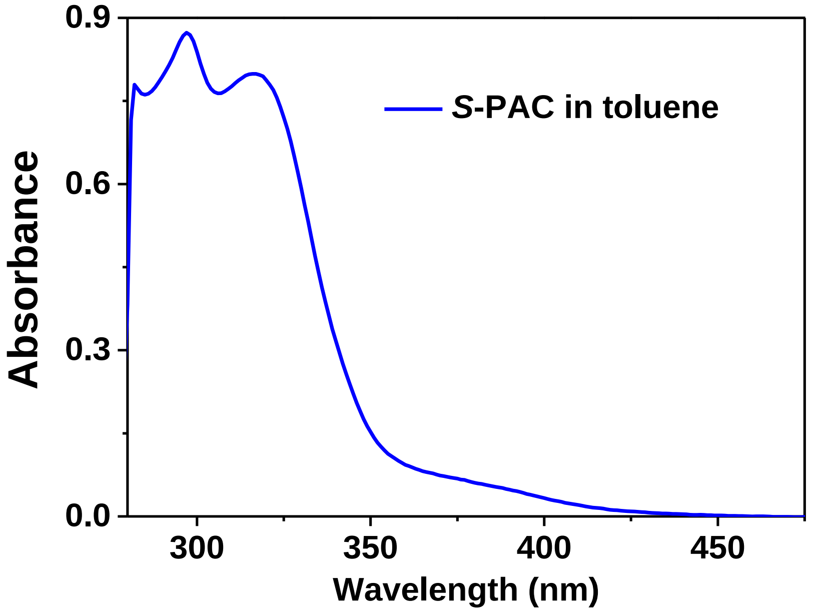

Figure S16. UV-Vis absorption of *S*-**PAC** spectra in toluene (c =1 × 10^{-5} M).

Table S4. The electrochemical properties of *R/S*-**PAC**

Samples	$E_{ox,oneset}$[a]	E_{HOMO}[b]	E_{LUMO}[c]	$E_{g,opt}$[d]
R-**PAC**	0.860 eV	−5.523 eV	−2.780 eV	2.743 eV
S-**PAC**	0.858 eV	−5.517 eV	−2.777 eV	2.740 eV

The onset of [a]oxidation curve; [b]$E_{HOMO} = -[E_{ox} - E_{(Fc/Fc+)} + 4.8]$ eV; [c]$E_{LUMO} = E_{HOMO} + E_g$; [d]$E_g$ = optical gap ($E_{g,opt} = 1240 / \lambda_{abs,onset}$).

7. Photophysical Properties

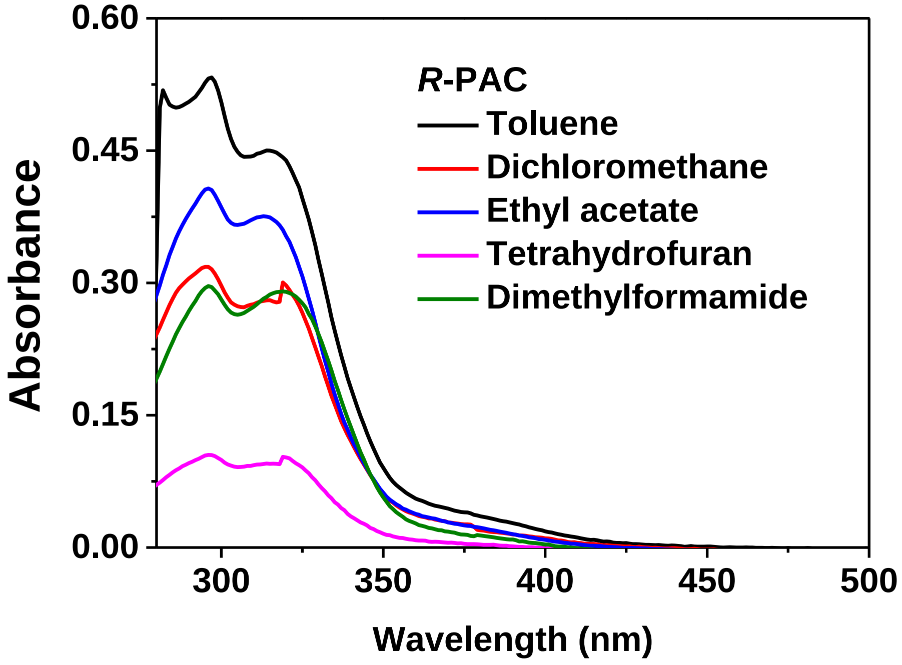

Figure S17. UV-Vis absorption spectra of *R*-**PAC** in various solvents (c = 1 × 10^{-5} M).

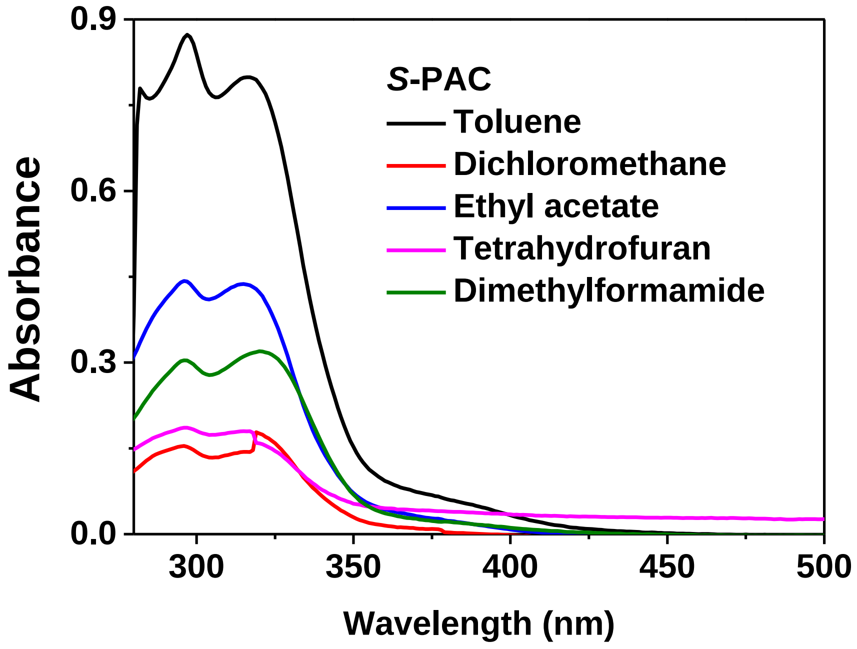

Figure S18. UV-Vis absorption spectra of *S*-**PAC** in various solvents (c = 1 × 10^{-5} M).

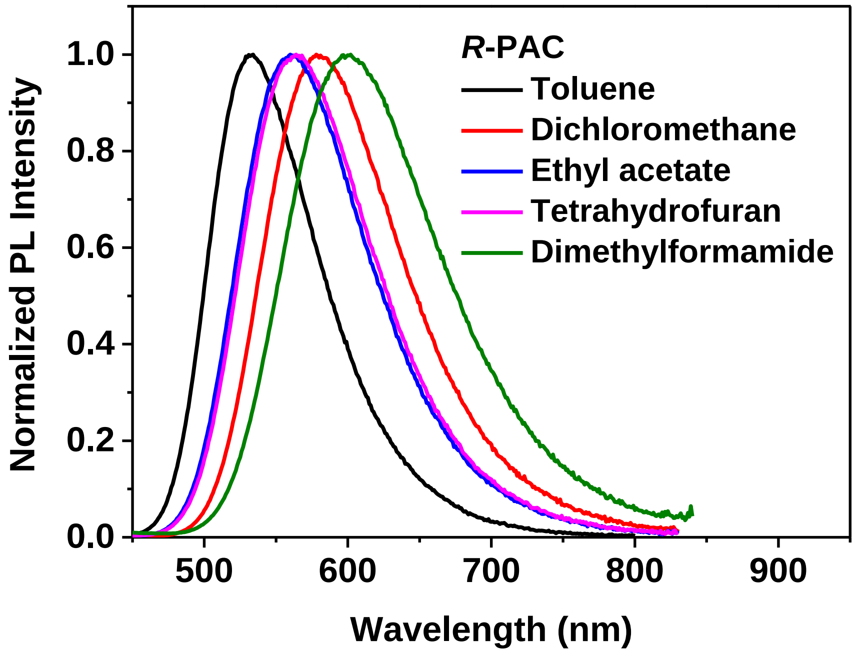

Figure S19. PL spectra of *R*-**PAC** in various solvents ($c = 1 \times 10^{-5}$ M).

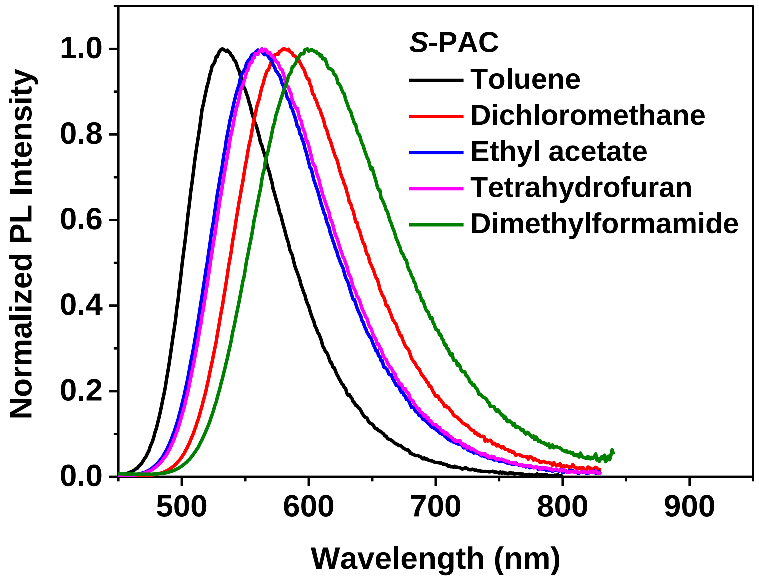

Figure S20. PL spectra of *S*-**PAC** in various solvents ($c = 1 \times 10^{-5}$ M).

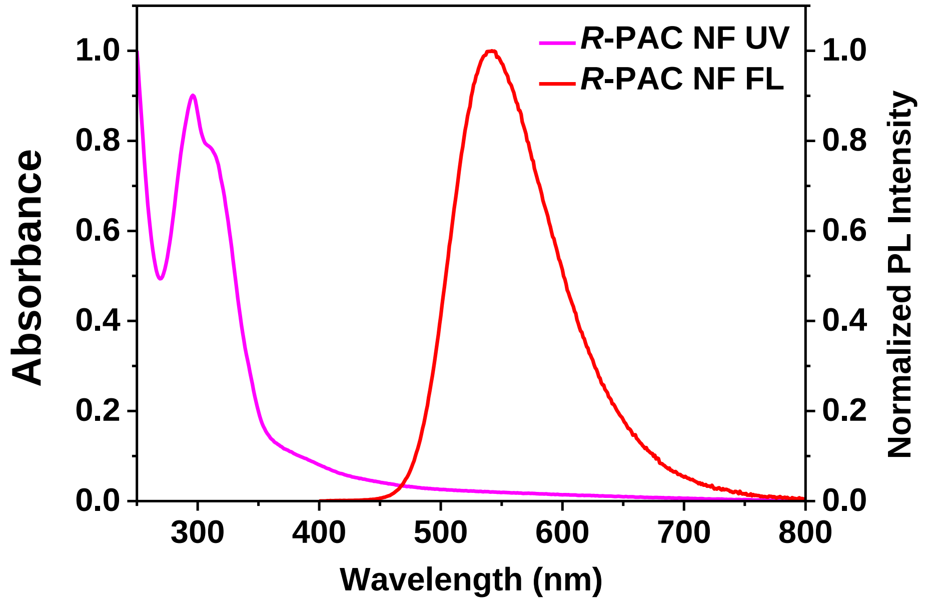

Figure S21. UV–vis absorption and fluorescence spectra of *R*-**PAC** at 298 K in neat film.

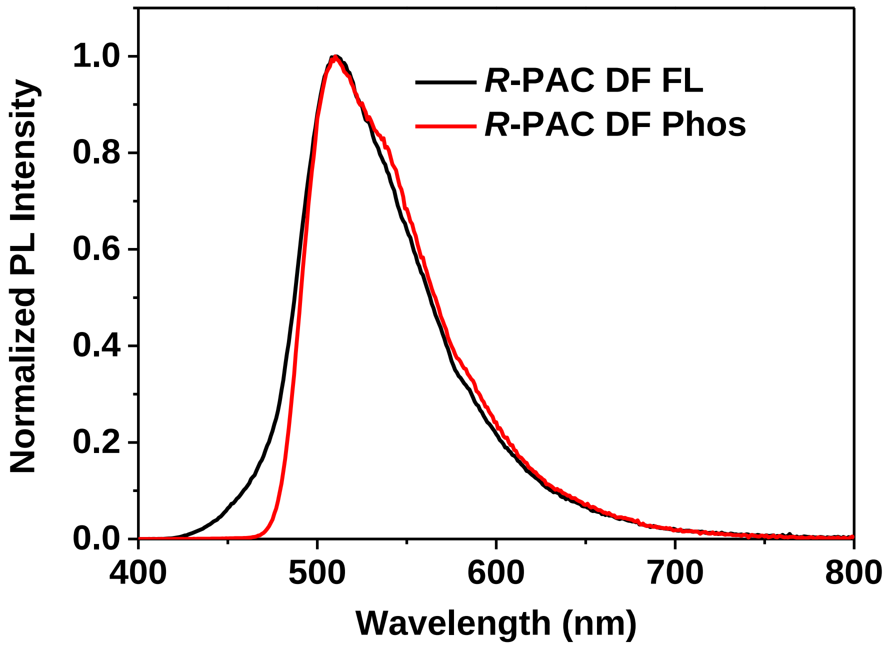

Figure S22. Fluorescence and phosphorescence spectra of *R*-**PAC** at 77 K in doped film.

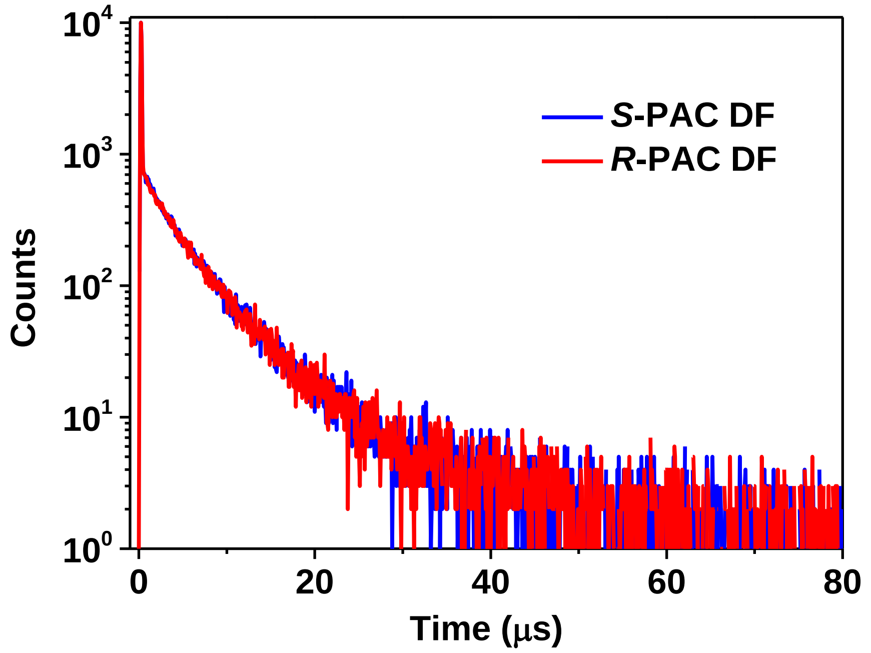

Figure S23. Transient photoluminescence (PL) decay curve of *R/S*-**PAC** at 298 K in doped film.

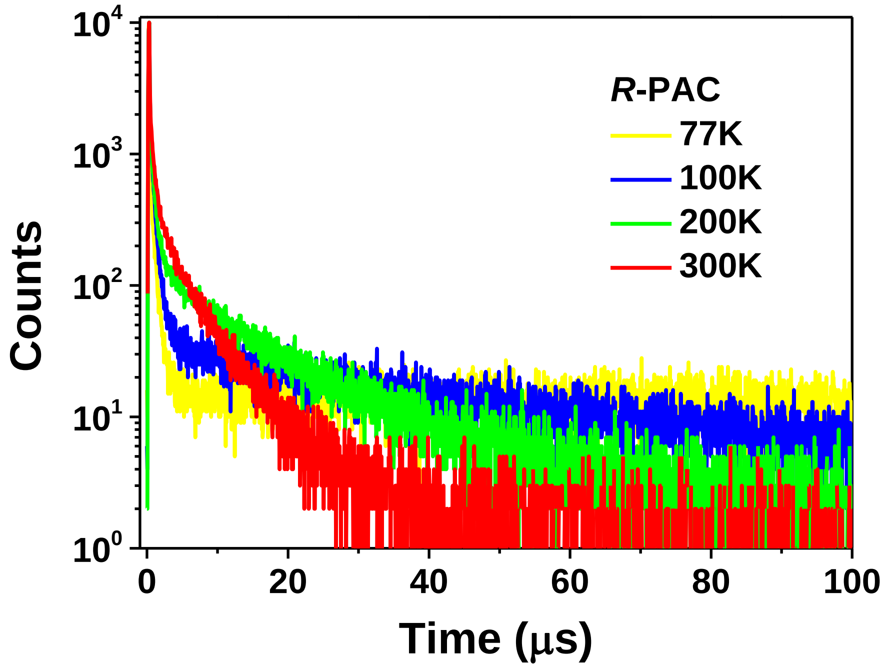

Figure S24. Temperature-dependent transient PL decay curves of *R*-**PAC** in doped film.

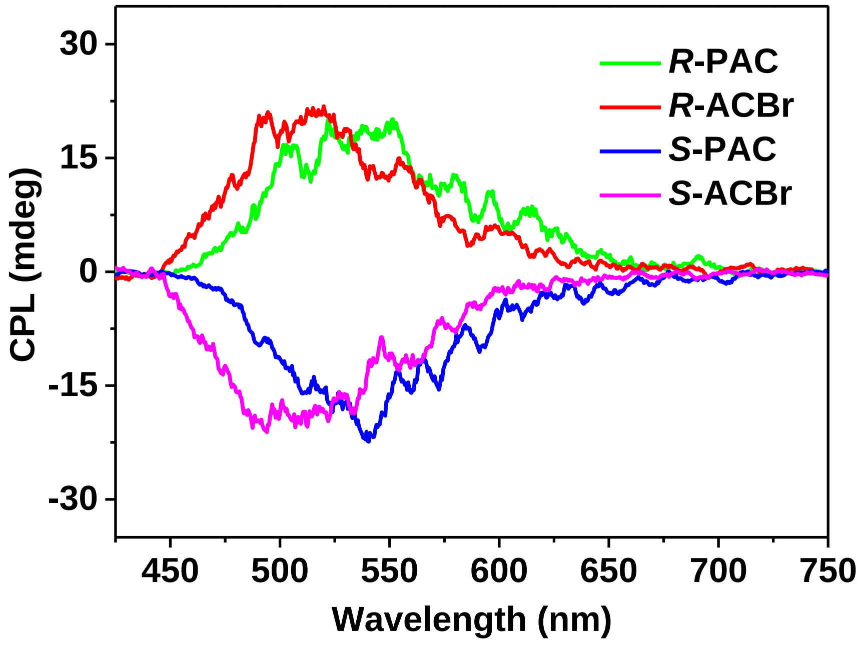

Figure S25. CPL spectra of *R/S*-**PAC** and *R/S*-**ACBr** in toluene solution (c = 10^{-5} M) at 298 K.

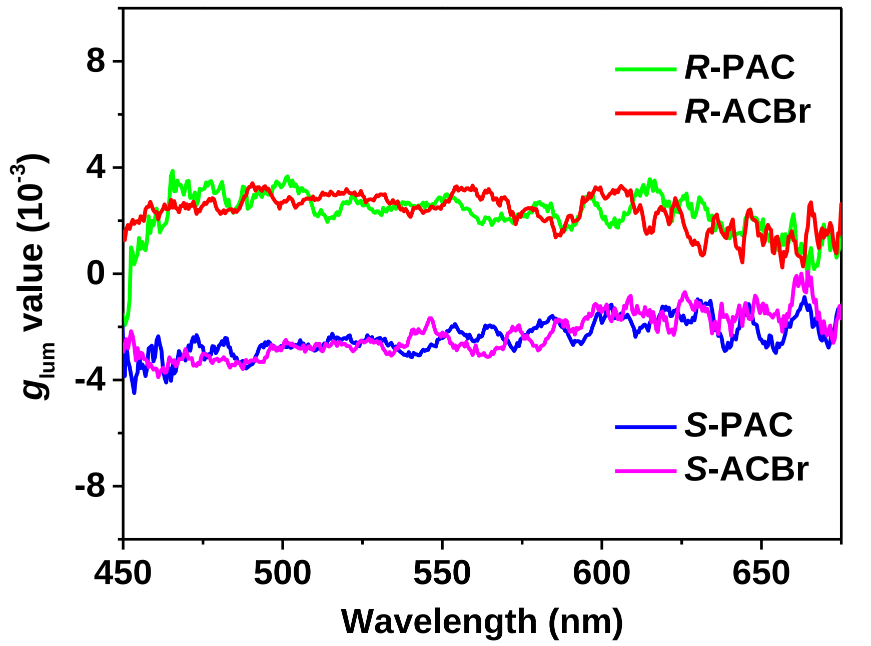

Figure S26. The g_{lum} values versus wavelength curves of *R/S*-**PAC** and *R/S*-**ACBr** in toluene solution (c = 10^{-5} M) at 298 K.

8. Device Fabrication and Characterization

Indium tin oxide (ITO) coated glass with a sheet resistance of 10 Ω per square was used as the anode substrate. Before the fabrication of devices, the ITO glass substrates were cleaned with Decon 90, rinsed in ultrapure water and ethanol, dried in an oven at 110 °C, and finally treated with O_2 plasma for two minutes to enhance the surface work function of ITO anode. Subsequently, 40 nm-thin PEDOT:PSS/PFI mixture was spin-coated on the ITO anode substrate as hole-injecting layer, then annealed at 120 °C for 15 min. Then, the solutions of TADF polymers (10 mg/mL in chlorobenzene) were spin-coated onto hole-injecting layer to form the emitting-layer, followed by annealing at 60 °C for 30 min. Finally, TPBi, LiF and Al were consecutively evaporated onto the emitting layer in a vacuum deposition chamber. The electroluminescence and current-voltage-luminance characteristics of the devices were measured with a computer-controlled Spectrascan PR 670 spectrophotometer and Keithley 2400 SourceMeter after device packaging.

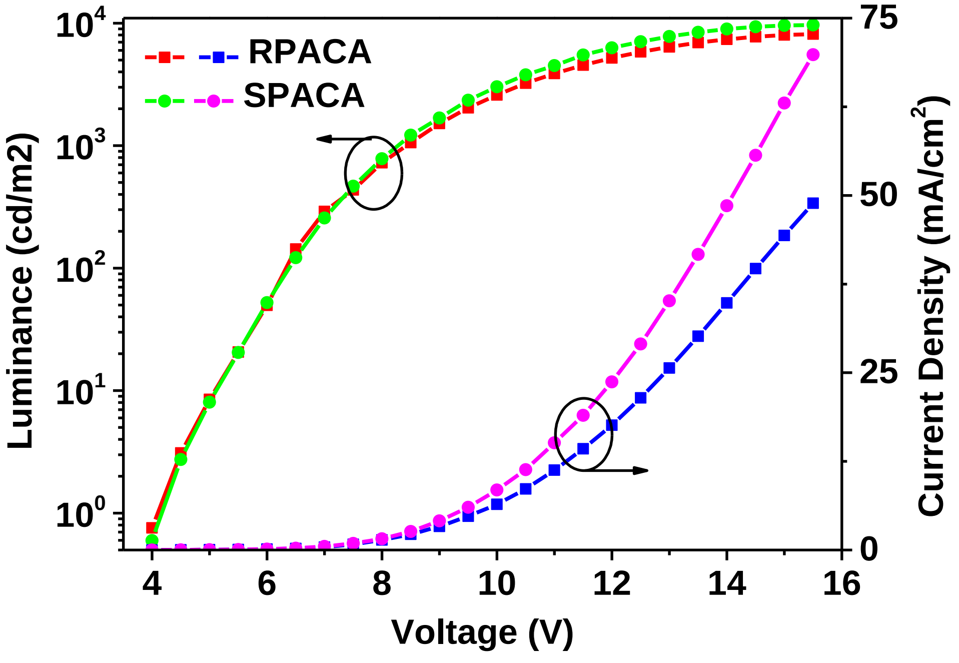

Figure S27. Luminance-voltage-current density (J-V-L) characteristics of RPACA and SPACA.

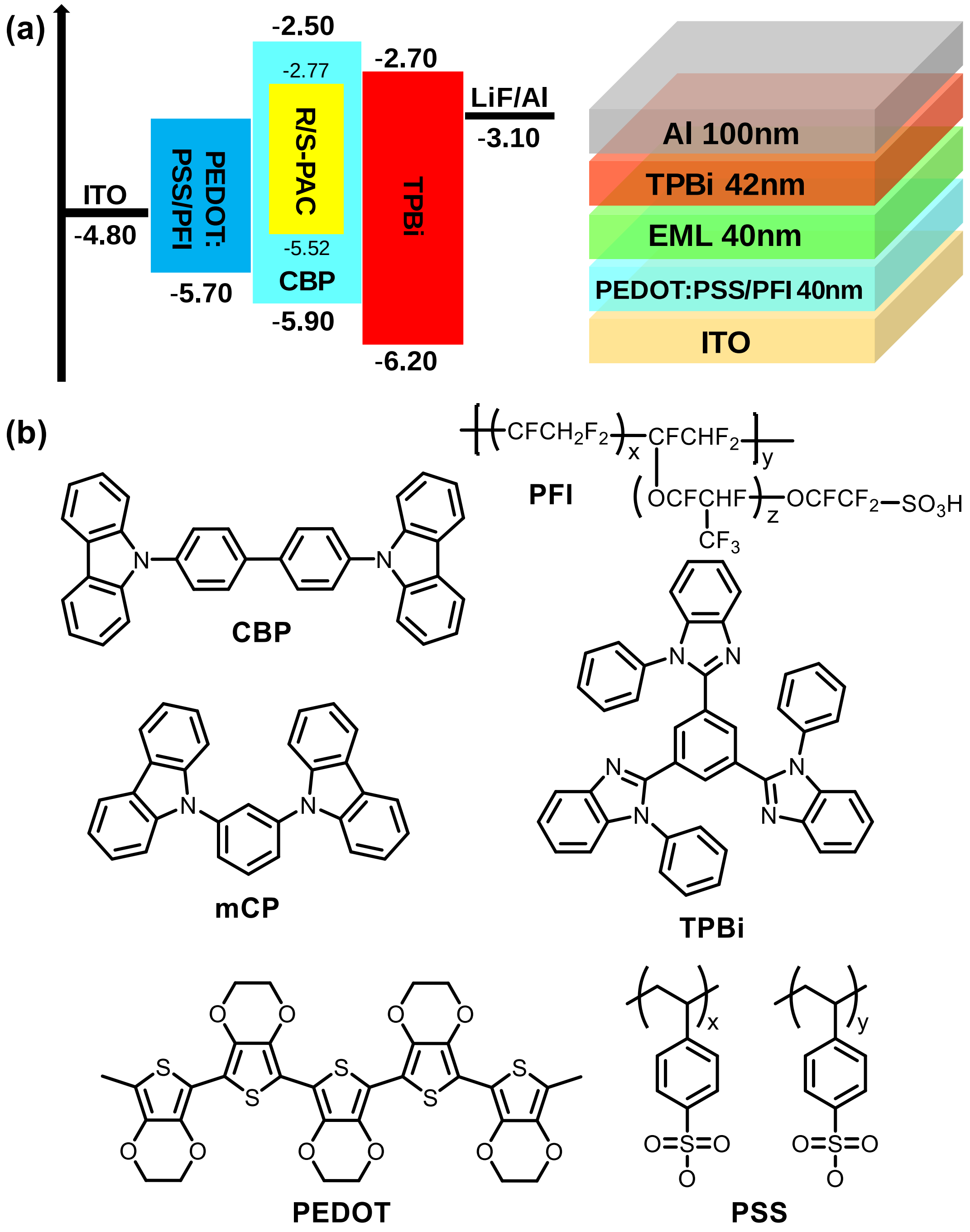

Figure S28. (a) Device structures and energy level diagrams of RPACB and SPACB. (b) molecular structures of used materials of devices.

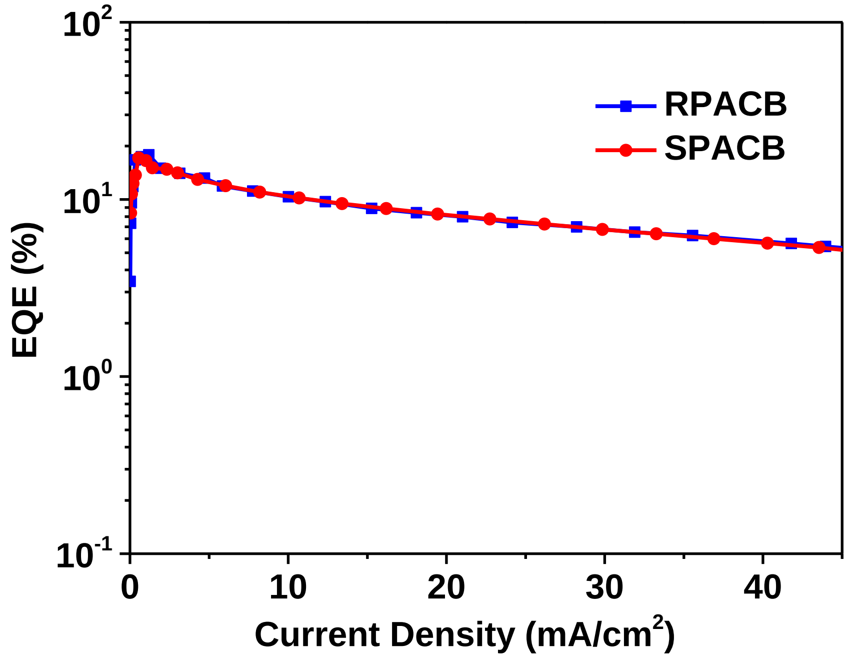

Figure S29. EQE-Current Density characteristics of RPACB and SPACB.

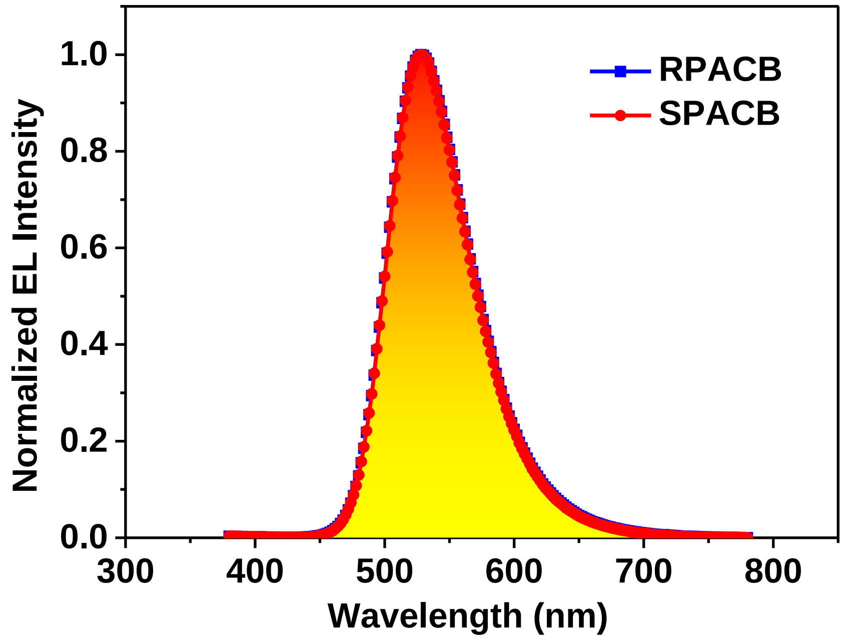

Figure S30. EL spectra of RPACB and SPACB at 6 V.

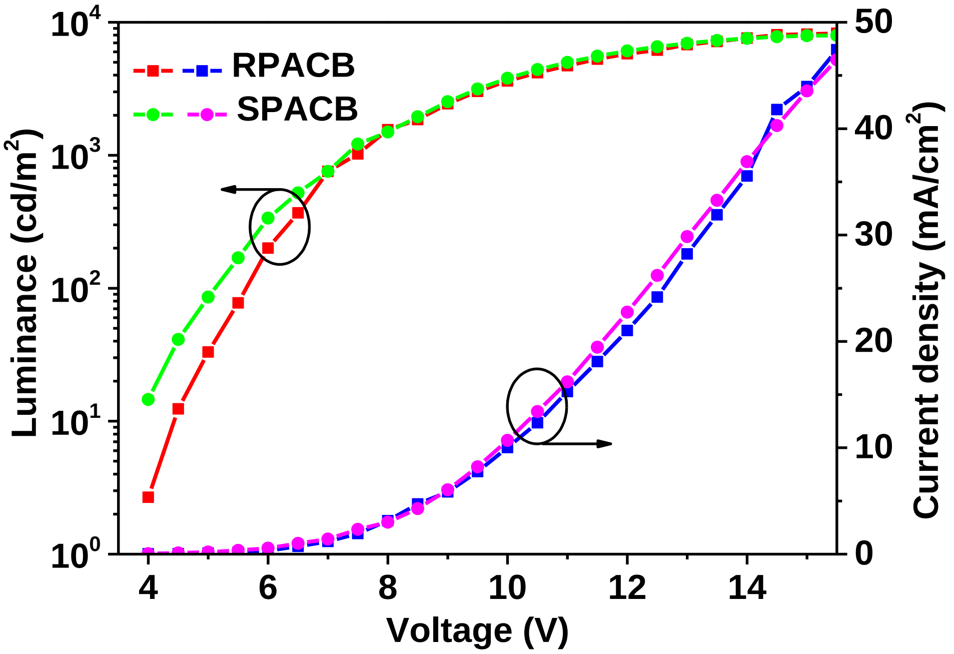

Figure S31. Luminance-voltage-current density (J-V-L) characteristics of RPACB and SPACB.

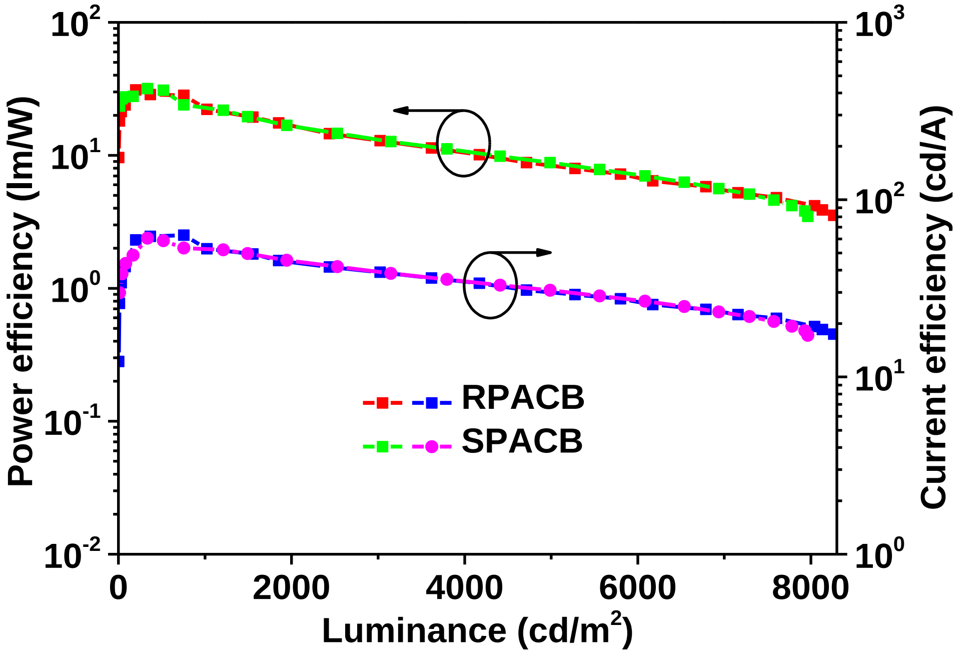

Figure S32. Power Efficiency-Luminance-Current Efficiency characteristics of RPACB and SPACB.

9. Copies of ^{1}H NMR and ^{13}C NMR Spectra of New Compounds

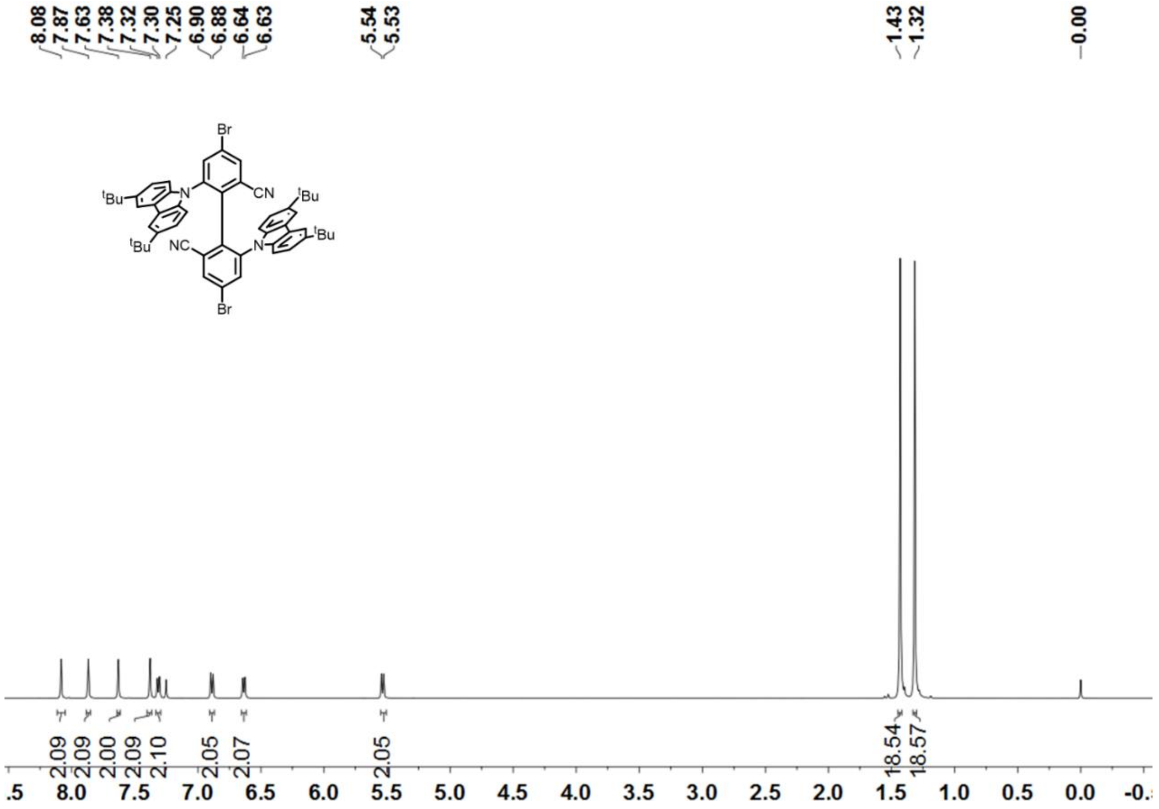

Figure S33. ^{1}H NMR spectrum (500 MHz, $CDCl_3$) of *rac*-**ACBr**.

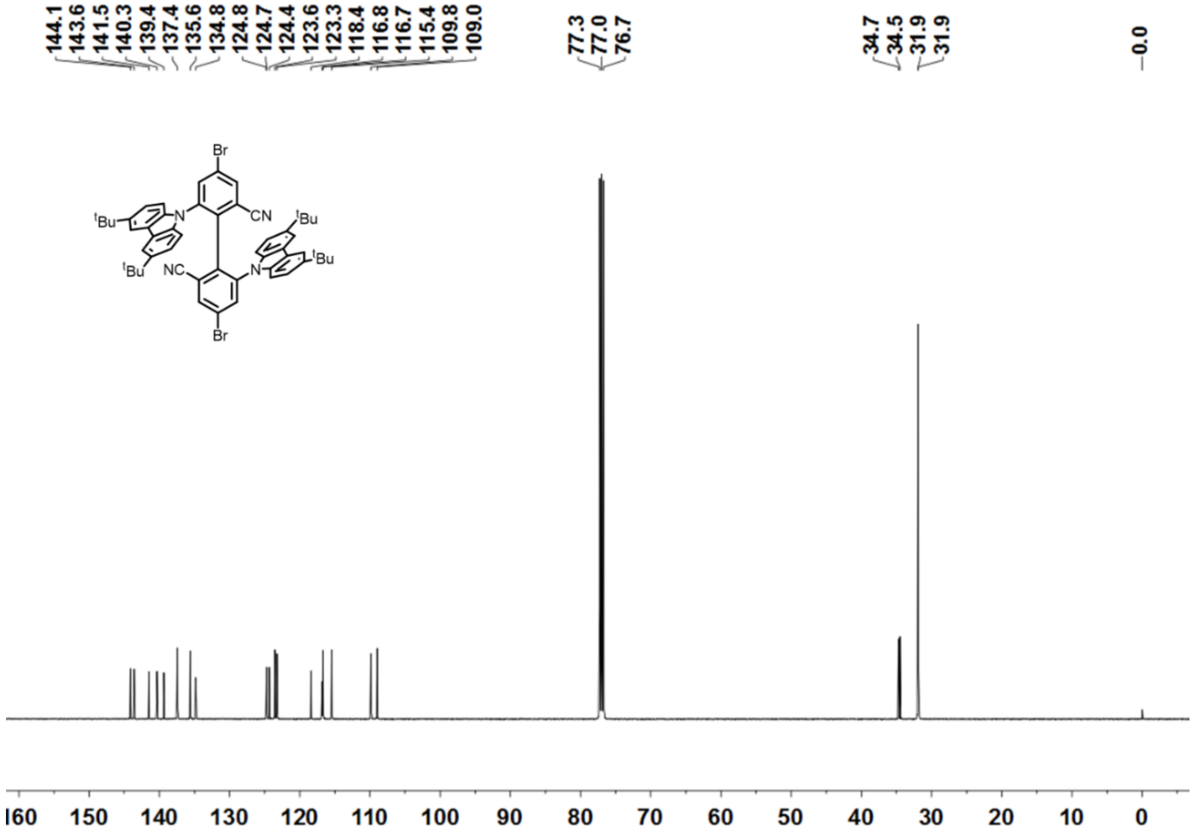

Figure S34. ^{13}C NMR spectrum (126 MHz, $CDCl_3$) of *rac*-**ACBr**.

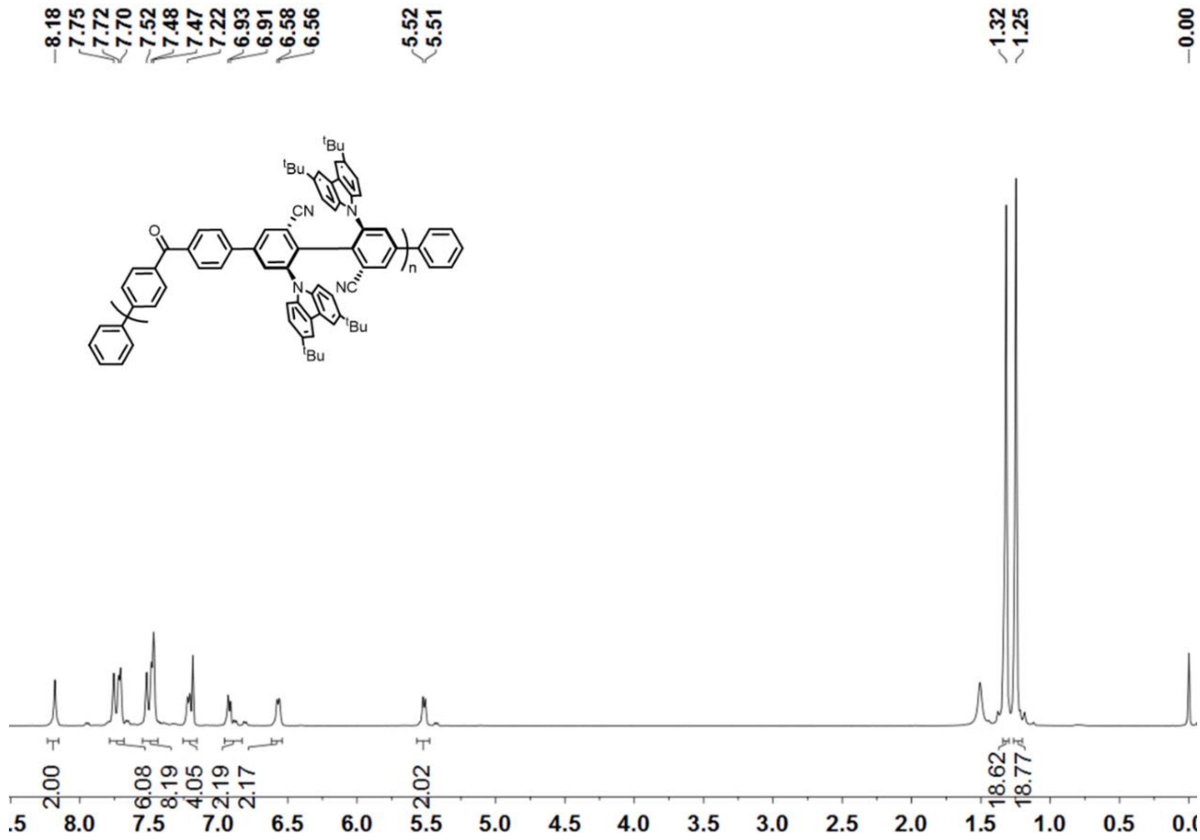

Figure S35. ^{1}H NMR spectrum (500 MHz, $CDCl_3$) of *R*-**PAC**.

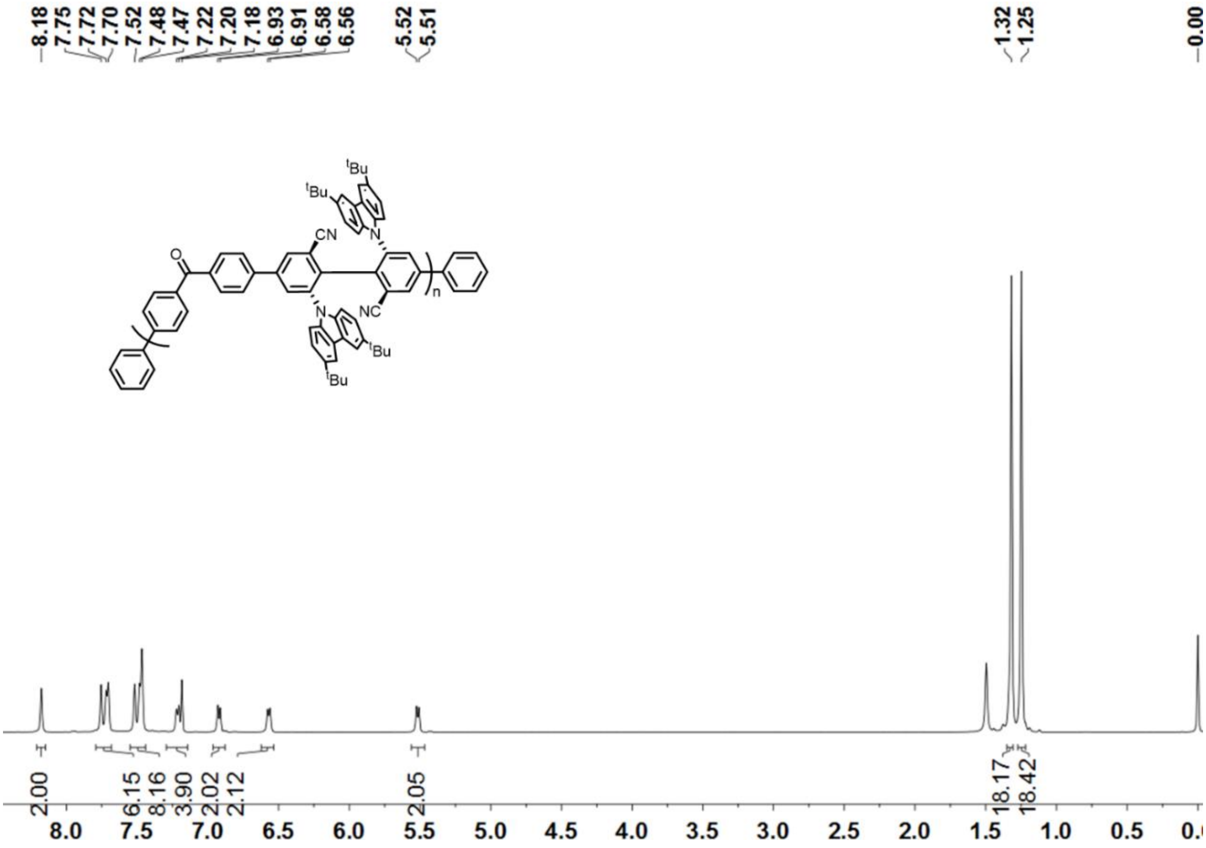

Figure S36. ^{1}H NMR spectrum (500 MHz, $CDCl_3$) of *S*-**PAC**.

Table S5. The device performances of the reported chiral conjugated polymer-based CP-OLEDs vs this work.

**Materials**	**Structure**	**Type of emitters**	**λ_{EL} (nm)**	**L_{max} (cd/m^2)**	**EQE_{max} (%)**	**g_{EL}**	**Ref**
**BMB-PPV-coBDMO-PPV**	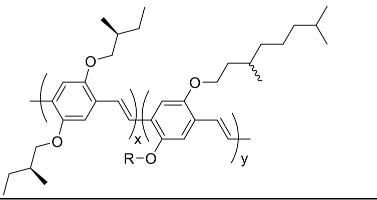	Common fluorescent polymer	600	-	-	-0.0017	[S3]
(*S*)-**PF4/1**	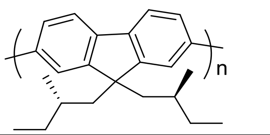	Common fluorescent polymer	425/ 512	-	-	+0.16	[S4] [S5]
(*S*)-**PF4/1-co-PF8**	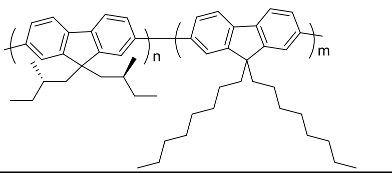			-	-	+0.05	
(*S*)-**PF8/1/1**	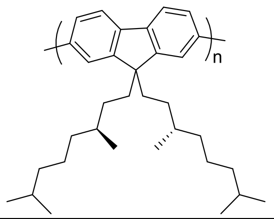			-	-	-0.25	
(*R*)-**PF2/6**	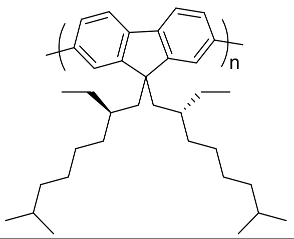			-	-	-0.25	
**c-PFBT**	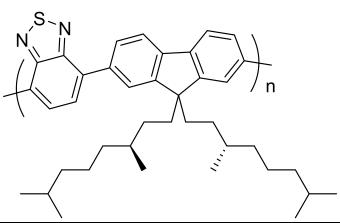	Common fluorescent polymer	510	80	-	-0.8	[S6]
S-/*R*-**P**	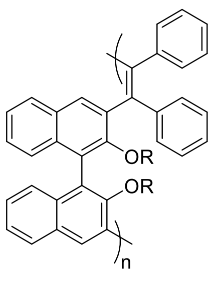 R=-C_8H_{17}-n	Common fluorescent polymer	505	1669/ 1270	-	+0.024/ -0.019	[S7]

**P2**	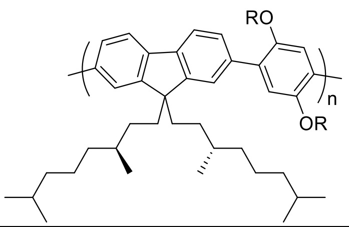	Common fluorescent polymer	400	-	-	-0.2	[S8]
R/S-**P2**	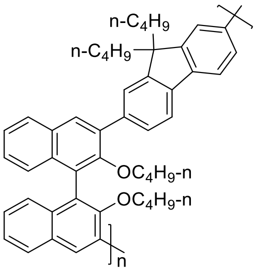	Common fluorescent polymer	418/ 418	205/ 201	0.012/ 0.010	-0.021/ +0.020	[S9]
R/S-**BP**	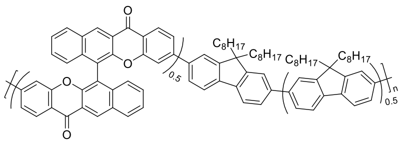	Common fluorescent polymer	(0.17, 0.21)/ (0.17, 0.21)	4107/ 4147	0.56/ 0.57	-0.0025/ +0.0023	
R/S-**WP1**	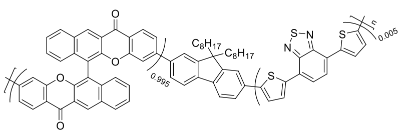	Common fluorescent polymer	(0.33, 0.28)/ (0.33, 0.27)	3503/ 3422	0.25/ 0.25	(-0.0011/- 0.0006)/ (+0.0009/ +0.0006)	[S10]
R/S-**WP2**	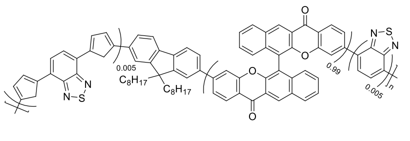	Common fluorescent polymer	(0.33, 0.34)/ (0.32, 0.33)	3013/ 3001	0.50/ 0.54	(-0.0018/- 0.0015)/- 0.0008 (+0.0011/ +0.0013/+ 0.0019)	
R/S-**P2G**	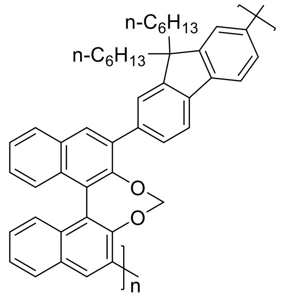	Common fluorescent polymer	489/ 489	2716/ 3018	0.18/ 0.21	-0.048/ +0.046	[S11]
R/S-**P**	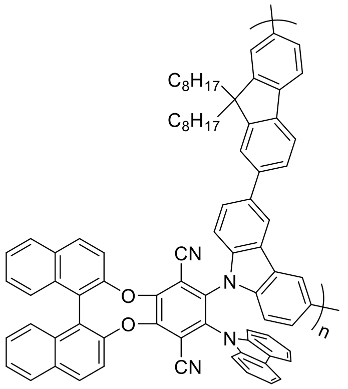	TADF polymer	546/ 544	8940/ 12180	14.9/ 15.8	-0.0015/ +0.0016	[S12]

R/S-**PT**	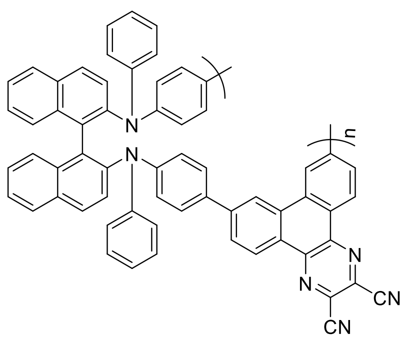	TADF polymer	662/ 662	826/ 743	6.2/ 5.8	-0.0016/ +0.0017	[S13]
R/S-**PAC**	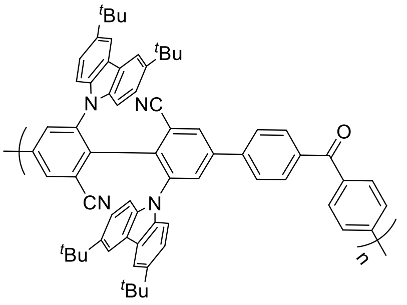	TADF polymer	524/ 524	8149/ 9661	17.8/ 17.1	+0.0032/ -0.0034	**This work**

10. References

[S1] Gaussian 16, Revision A.03, M. J. Frisch, G. W. Trucks, H. B. Schlegel, G. E. Scuseria, M. A. Robb, J. R. Cheeseman, G. Scalmani, V. Barone, G. A. Petersson, H. Nakatsuji, X. Li, M. Caricato, A. V. Marenich, J. Bloino, B. G. Janesko, R. Gomperts, B. Mennucci, H. P. Hratchian, J. V. Ortiz, A. F. Izmaylov, J. L. Sonnenberg, D. Williams-Young, F. Ding, F. Lipparini, F. Egidi, J. Goings, B. Peng, A. Petrone, T. Henderson, D. Ranasinghe, V. G. Zakrzewski, J. Gao, N. Rega, G. Zheng, W. Liang, M. Hada, M. Ehara, K. Toyota, R. Fukuda, J. Hasegawa, M. Ishida, T. Nakajima, Y. Honda, O. Kitao, H. Nakai, T. Vreven, K. Throssell, J. A. Montgomery, Jr., J. E. Peralta, F. Ogliaro, M. J. Bearpark, J. J. Heyd, E. N. Brothers, K. N. Kudin, V. N. Staroverov, T. A. Keith, R. Kobayashi, J. Normand, K. Raghavachari, A. P. Rendell, J. C. Burant, S. S. Iyengar, J. Tomasi, M. Cossi, J. M. Millam, M. Klene, C. Adamo, R. Cammi, J. W. Ochterski, R. L. Martin, K. Morokuma, O. Farkas, J. B. Foresman, and D. J. Fox, Gaussian, Inc., Wallingford CT, **2016**.

[S2] W. Humphrey, A. Dalke, K. Schulten, *J. Mol. Graphics* **1996**, *14*, 33.

[S3] E. Peeters, M. P. T. Christiaans, R. A. J. Janssen, H. F. M. Schoo, H. P. J. M. Dekkers, E. W. Meijer, *J. Am. Chem. Soc.* **1997**, *119*, 9909.

[S4] M. Oda, H. Nothofer, G. Lieser, U. Scherf, S. C. J. Meskers, *Adv. Mater.* **2000**, *12*, 362.

[S5] M. Oda, H. Nothofer, U. Scherf, V. Šunjić, D. Richter, W. Regenstein, D. Neher, *Macromolecules* **2002**, *35*, 6792.

[S6] D. D. Nuzzo, C. Kulkarni, B. Zhao, E. Smolinsky, F. Tassinari, S. C. J. Meskers, R. Naaman, E. W. Meijer, R. H. Friend, *ACS Nano* **2017**, *11*, 12713.

[S7] L. Yang, Y. Zhang, X. Zhang, N. Li, Y. Quan, Y. Cheng, *Chem. Commun.* **2018**, *54*, 9663.

[S8] C. Kulkarni, M. H. C. van Son, D. Di Nuzzo, S. C. J. Meskers, A. R. A. Palmans, E. W. Meijer, *Chem. Mater.* 2019, 31, 6633.

[S9] Z. Geng, Y. Zhang, Y. Zhang, Y. Li, Y. Quan, Y. Cheng, *J. Mater. Chem. C* **2021**, *9*, 12141.

[S10] Y. Zhang, T. Jing, Y. Quan, S. Ye, Y. Cheng, *Adv. Optical Mater.* **2022**, *10*, 2200915.

[S11] Z. Geng, Y. Zhang, Y. Zhang, Y. Quan, Y. Cheng, *Angew. Chem. Int. Ed.* **2022**, *61*, e202202718.

[S12] J.-M. Teng, D.-W. Zhang, Y.-F. Wang, C.-F. Chen, *ACS Appl. Mater. Interfaces* **2022**, *14*, 1578.

[S13] J.-M. Teng, C.-F. Chen, *Adv. Optical Mater.* **2023**, *11*, 2300550.
