# Supplementary material for: Acceptor Copolymerized Axially Chiral Conjugated Polymers with TADF Properties for Efficient Circularly Polarized Electroluminescence
Source: Adv Sci (Weinh). 2024 Mar 29;11(23):2309031. doi: 10.1002/advs.202309031 (PMC11186117; doi:10.1002/advs.202309031)

checkCIF/PLATON report

Structure factors have been supplied for datablock(s) tx12964_sq

THIS REPORT IS FOR GUIDANCE ONLY. IF USED AS PART OF A REVIEW PROCEDURE FOR PUBLICATION, IT SHOULD NOT REPLACE THE EXPERTISE OF AN EXPERIENCED CRYSTALLOGRAPHIC REFEREE.

No syntax errors found. CIF dictionary Interpreting this report

Datablock: tx12964_sq

```
Bond precision:   C-C = 0.0062 A          Wavelength=1.54184

Cell:             a=12.4756(1)     b=15.6189(2)       c=12.9645(2)
                  alpha=90         beta=95.102(1)     gamma=90
Temperature:      170 K

                 Calculated                     Reported
Volume           2516.19(5)                     2516.19(5)
Space group      P 21                           P 1 21 1
Hall group       P 2yb                          P 2yb
Moiety formula   C54 H52 Br2 N4 [+ solvent]     C54 H52 Br2 N4
Sum formula      C54 H52 Br2 N4 [+ solvent]     C54 H52 Br2 N4
Mr               916.80                         916.81
Dx,g cm-3        1.210                          1.210
Z                2                              2
Mu (mm-1)        2.319                          2.319
F000             948.0                          948.0
F000'            947.36
h,k,lmax         15,19,16                       15,19,16
Nref             10440[ 5420]                   10102
Tmin,Tmax        0.895,0.911                    0.249,1.000
Tmin'            0.870

Correction method= # Reported T Limits: Tmin=0.249 Tmax=1.000
AbsCorr = MULTI-SCAN

Data completeness= 1.86/0.97       Theta(max)= 75.519

R(reflections)= 0.0449( 9915)                          wR2(reflections)=
                                                       0.1236( 10102)
S = 1.029                   Npar= 554
```

The following ALERTS were generated. Each ALERT has the format
test-name_ALERT_alert-type_alert-level.
Click on the hyperlinks for more details of the test.

Alert level B

```
PLAT230_ALERT_2_B Hirshfeld Test Diff for    C24      --C25      .         7.5 s.u.
```

Alert level C

```
PLAT213_ALERT_2_C Atom Br2             has ADP max/min Ratio .....         3.3 prolat
PLAT213_ALERT_2_C Atom C26             has ADP max/min Ratio .....         3.2 prolat
PLAT220_ALERT_2_C NonSolvent   Resd 1  C    Ueq(max)/Ueq(min) Range        5.2 Ratio
PLAT220_ALERT_2_C NonSolvent   Resd 1  N    Ueq(max)/Ueq(min) Range        3.4 Ratio
PLAT222_ALERT_3_C NonSolvent Resd 1  H   Uiso(max)/Uiso(min) Range         5.6 Ratio
PLAT242_ALERT_2_C Low    'MainMol' Ueq as Compared to Neighbors of         C20 Check
PLAT242_ALERT_2_C Low    'MainMol' Ueq as Compared to Neighbors of         C47 Check
PLAT341_ALERT_3_C Low Bond Precision on  C-C Bonds ..............      0.00616 Ang.
```

Alert level G

```
PLAT431_ALERT_2_G Short Inter HL..A Contact  Br2      ..N1       .        3.26 Ang.
                                               2-x,1/2+y,-z  =      2_755 Check
PLAT605_ALERT_4_G Largest Solvent Accessible VOID in the Structure         127 A**3
PLAT869_ALERT_4_G ALERTS Related to the Use of SQUEEZE  Suppressed           ! Info
PLAT883_ALERT_1_G No Info/Value for _atom_sites_solution_primary .     Please Do !
PLAT912_ALERT_4_G Missing # of FCF Reflections Above STh/L=  0.600          77 Note
PLAT913_ALERT_3_G Missing # of Very Strong Reflections in FCF ....           1 Note
PLAT978_ALERT_2_G Number C-C Bonds with Positive Residual Density.           4 Info
```

0 **ALERT level A** = Most likely a serious problem - resolve or explain
1 **ALERT level B** = A potentially serious problem, consider carefully
8 **ALERT level C** = Check. Ensure it is not caused by an omission or oversight
7 **ALERT level G** = General information/check it is not something unexpected

1 ALERT type 1 CIF construction/syntax error, inconsistent or missing data
9 ALERT type 2 Indicator that the structure model may be wrong or deficient
3 ALERT type 3 Indicator that the structure quality may be low
3 ALERT type 4 Improvement, methodology, query or suggestion
0 ALERT type 5 Informative message, check

It is advisable to attempt to resolve as many as possible of the alerts in all categories. Often the minor alerts point to easily fixed oversights, errors and omissions in your CIF or refinement strategy, so attention to these fine details can be worthwhile. In order to resolve some of the more serious problems it may be necessary to carry out additional measurements or structure refinements. However, the purpose of your study may justify the reported deviations and the more serious of these should normally be commented upon in the discussion or experimental section of a paper or in the "special_details" fields of the CIF. checkCIF was carefully designed to identify outliers and unusual parameters, but every test has its limitations and alerts that are not important in a particular case may appear. Conversely, the absence of alerts does not guarantee there are no aspects of the results needing attention. It is up to the individual to critically assess their own results and, if necessary, seek expert advice.

Publication of your CIF in IUCr journals

A basic structural check has been run on your CIF. These basic checks will be run on all CIFs submitted for publication in IUCr journals (*Acta Crystallographica*, *Journal of Applied Crystallography*, *Journal of Synchrotron Radiation*); however, if you intend to submit to *Acta Crystallographica Section C* or *E* or *IUCrData*, you should make sure that full publication checks are run on the final version of your CIF prior to submission.

Publication of your CIF in other journals

Please refer to the *Notes for Authors* of the relevant journal for any special instructions relating to CIF submission.

PLATON version of 10/05/2023; check.def file version of 10/05/2023

Datablock tx12964_sq - ellipsoid plot

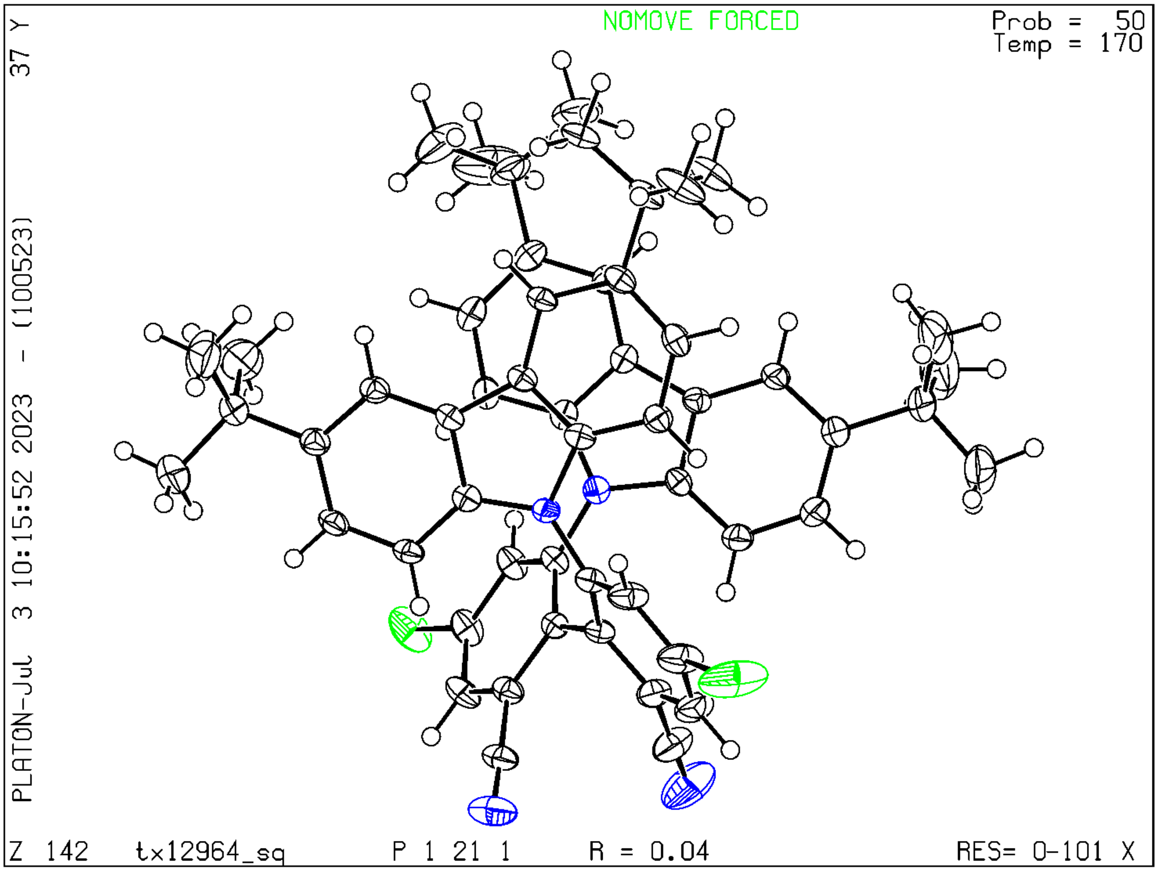

Supplement: Supplementary file 2 — Supporting Information [file ADVS-11-2309031-s002.zip › checkcif-S-ACBr.pdf]
